# Mechanism of the Gut Microbiota Colonization Resistance and Enteric Pathogen Infection

**DOI:** 10.3389/fcimb.2021.716299

**Published:** 2021-12-23

**Authors:** Israr Khan, Yanrui Bai, Lajia Zha, Naeem Ullah, Habib Ullah, Syed Rafiq Hussain Shah, Hui Sun, Chunjiang Zhang

**Affiliations:** ^1^ School of Life Sciences, Lanzhou University, Lanzhou, China; ^2^ Key Laboratory of Cell Activities and Stress Adaptations, Ministry of Education, Lanzhou University, Lanzhou, China; ^3^ Gansu Key Laboratory of Biomonitoring and Bioremediation for Environmental Pollution, Lanzhou University, Lanzhou, China; ^4^ Gansu Key Laboratory of Functional Genomics and Molecular Diagnosis, Lanzhou University, Lanzhou, China; ^5^ Cuiying Biomedical Research Centre, Lanzhou University Second Hospital, Lanzhou, China; ^6^ Department of Microecology, School of Basic Medical Sciences, Dalian Medical University, Dalian, China

**Keywords:** gut microbiota, colonization resistance, commensals, pathogens, microbial interaction, enteric infections

## Abstract

The mammalian gut microbial community, known as the gut microbiota, comprises trillions of bacteria, which co-evolved with the host and has an important role in a variety of host functions that include nutrient acquisition, metabolism, and immunity development, and more importantly, it plays a critical role in the protection of the host from enteric infections associated with exogenous pathogens or indigenous pathobiont outgrowth that may result from healthy gut microbial community disruption. Microbiota evolves complex mechanisms to restrain pathogen growth, which included nutrient competition, competitive metabolic interactions, niche exclusion, and induction of host immune response, which are collectively termed colonization resistance. On the other hand, pathogens have also developed counterstrategies to expand their population and enhance their virulence to cope with the gut microbiota colonization resistance and cause infection. This review summarizes the available literature on the complex relationship occurring between the intestinal microbiota and enteric pathogens, describing how the gut microbiota can mediate colonization resistance against bacterial enteric infections and how bacterial enteropathogens can overcome this resistance as well as how the understanding of this complex interaction can inform future therapies against infectious diseases.

## Introduction

The resident microbes of the human gut, collectively termed as gut microbiota (Sender et al., 2016), are a highly dynamic and diverse ecosystem, estimated to be composed of trillions of microbial cells, which approximately outnumber by a ratio of 10.1 or roughly equivalent to the number of cells in the human body and encode 500 times more genes than the human genome (Li et al., 2014; Sender et al., 2016; Tierney et al., 2019; Koh and Bäckhed, 2020). Microbial density distribution across the gastrointestinal tract (GIT) is variable from the upper proximal to the distal end of the intestine, mainly dominated by obligate anaerobic bacteria (Sekirov et al., 2010; The Human Microbiome Project Consortium, 2012). The normal average healthy gut microbiome is not defined yet; however, it is generally characterized by the presence of high diversity and richness of beneficial bacteria and a lower number of pathogenic bacteria/pathobionts in a healthy state of the host (Raman et al., 2005; Huttenhower et al., 2012). The normal gut microbial community as a whole behaves as commensals that contribute to the host in a multitude of essential functions; therefore, it is generally referred to as the commensal microbiota (Kamada et al., 2013). Commonly, the gut microbiota in healthy individuals is populated with five major phyla, namely, Firmicutes, Bacteroidetes, Actinobacteria, Verrucomicrobia, and Proteobacteria, although there is a considerable variation in the diversity and relative abundance at the lower taxonomic level; consequently, the gut microbiota of each individual is unique at the genus and species levels (Raman et al., 2005; Schroeder and Bäckhed, 2016). It has been known for years that the gut microbiota has co-evolved with the host, where the host provides a stable habitat to the microbes. In return, microbes benefit the host with many physiological processes such as food digestion and absorption *via* production of hydrolytic enzymes and co-factor molecules such as vitamin production, which are critical for the health of the host (Koh et al., 2016; Martinez-Guryn et al., 2018). Recently, the gut microbiota has been recognized in the more complex biological processes of the host such as metabolism, regulation of the gut barrier function, and immunity development. In addition, the more important function imposed by the gut microbiota is resistance against pathogens, protecting the host from pathogen infections, a phenomenon at present termed as colonization resistance (Kamada et al., 2013; Lawley and Walker, 2013; Sonnenburg and Bäckhed, 2016; Scott et al., 2020; Zheng et al., 2020). On the other hand, the altered gut microbiota has also been attributed to a variety of disease pathologies from intestinal functional to systemic metabolic diseases as well as in pathogen infections (Bohnhoff et al., 1954; Theriot et al., 2014; McKenney and Pamer, 2015; Blander et al., 2017; Fan and Pedersen, 2021). The altered gut composition during disease is recognized to have an excessive number of pathogenic bacteria/pathobiont members and a lower number of commensals (Theriot et al., 2014; Abt et al., 2016; Gagliardi et al., 2018; Khan et al., 2019), indicating that gut microbiota composition may have a role in disease pathogenesis as well as in host susceptibility to disease risk and outcomes; therefore, in the current microbiome research, the gut microbiota is considered as a moderator in host health and disease (Feng et al., 2018). Recently, the research on gut microbiome focuses more on the gut microbiota involvement in the pathogenesis of metabolic (obesity, diabetes, NAFLD), chronic immune [inflammatory bowel disease (IBD), arthritis, CNS inflammation], and cancerous diseases and revealed a microbe–host or microbe–environment interaction in disease initiation and progression (which is out of the scope of this review). However, little attention has been given to the role of gut microbiota in pathogen infections to explore the microbe–microbe interaction in the gut microbial community. Growing evidence shows that the expansion of the enteric bacterial infections may be associated with the loss of gut microbiota colonization resistance that prevents the overgrowth of resident pathobionts and the entry of exogenous obligate pathogens under the homeostatic condition (Bohnhoff et al., 1954; Theriot et al., 2014; McKenney and Pamer, 2015).

Colonization resistance is a phenomenon whereby the normal gut microbiota resists the invasion of the exogenous pathogens and the expansion of the resident pathobionts (Lawley and Walker, 2013). This notion is well supported by the induction of severe infections by enteric bacterial pathogens in germ-free or antibiotic-treated mice compared with conventionally raised or untreated control mice upon pathogenic bacteria inoculation (Sprinz et al., 1961; Waaij et al., 1971; Sekirov et al., 2008; Lawley et al., 2009) as well as with the treatment of bacterial infection models by fecal microbiota transplantation or probiotic gut microbiota species administration (Fukuda et al., 2011; Fukuda et al., 2012; Nood et al., 2013), indicating that the normal resident gut microbes play a central role in the prevention of pathogen colonization in the gut to cause intestinal infection. The mechanisms through which the intestinal microbiota provide colonization resistance are complex and have not been fully described; however, many involve direct interactions (such as nutrient competition, niche exclusion, toxic substances, and metabolite production) between bacterial communities (commensals–pathogens/pathobionts), and others act by indirect mechanisms that modulate the host system physiology, particularly the host immune response. Together, these mechanisms impart to colonization resistance against exogenous pathogenic microorganisms and resident pathobionts in the gut environment (Rolhion and Chassaing, 2016; Sorbara and Pamer, 2019). However, the high incidence of enteric infections caused by bacterial pathogens indicates that microbiota-mediated colonization resistance can be distressed and turn ineffective. Various factors such as host genetics, diet, and antibiotic usage that can alter the composition and functional capacity of the gut microbial community affect colonization resistance (Bokulich et al., 2016; Lim et al., 2017; Ducarmon et al., 2019; Pickard and Núñez, 2019). The disturbance in colonization resistance causes an outgrowth of opportunistic bacterial species that are typically present in low to very high numbers, which can harm the host, such as members of the Enterobacteriaceae family, as well as colonization by pathogenic bacteria, such as *Clostridium difficile* and *Salmonella enterica* serovar Typhimurium (*S.* Typhimurium) (Theriot and Young, 2015; Rivera-Chávez and Bäumler, 2015; Abt et al., 2016), thereby rendering opportunities for pathogens to utilize disruption in colonization resistance and colonize the gut, which ultimately leads to cause infection.

## Gut Microbiota in Diseases

In a healthy host, gut commensals are dominant over pathobionts, while an imbalance is shown in their composition with intestinal functional and infectious diseases, where the pathogenic counterpart dominates over commensals (Willing et al., 2009; Li et al., 2012; Oh et al., 2012; Jenior et al., 2018; Lv et al., 2019). The outcompeting of pathogens during the normal physiological condition (Becattini et al., 2017; Thanissery et al., 2017; Jacobson et al., 2018) and the expansion of pathogens (Rivera-Chávez et al., 2016) during disease condition reflect a kind of interaction phenomenon between commensals and pathogenic microbes. During disease, alteration in the homeostatic gut microbiota occurs either due to changes in the host factors (gene expression, immunity such as inflammation) or environmental factors (diet or antibiotic). As a result, alterations occur in the physiological environment (pH) and in the metabolic and nutritional landscape of the intestine, which may favor the growth of the pathogenic microbes and inhibit commensals that may increase the risk of pathogen colonization and infections (Stecher et al., 2007; Theriot et al., 2014). The altered gut microbial community dominated with pathogenic bacteria further aggravates the gut condition by inducing intestinal inflammation, which causes further enhancement in pathogen growth, virulence, and survival maintenance (Baümler and Sperandio, 2016; Ducarmon et al., 2019). The growing power of innovative computational analysis, multi-omics data analysis technologies (metagenomics, transcriptomics, and metabolomics), and the use of more conventional study approaches expanded our knowledge on the gut microbiota interactions and their impact on the metabolic and physiological landscape of the intestine in relevance to the severity and outcome of gastrointestinal infections.

With the recent advancements in the field of microbiome studies, the current research is focused on the associations between the microbiota, host, and pathogenic bacteria to unravel how the composition of the microbiota can offer either resistance or assistance to the invading pathogenic/pathobiont species. The majority of these studies were conducted in the GIT, in which associations between the host and microbes are of paramount importance. The gut microbiota, commensals, and pathogenic bacteria are adapted to the gut environment and establish complex ecological networks within the community and with the host to acquire their needs such as nutrients and maintain the normal gut microbial composition and survival. The gut microbial community establishes symbiotic relationships with commensal members to survive and remain dominant over pathogens. For example, in the gut microbiota, certain commensal species such as *Lactobacillus* spp. and *Eubacterium dolichum* are unable to manufacture certain amino acids and thus obtain these critical molecules from the host gut lumen contents or habitat (Pridmore et al., 2004; Turnbaugh et al., 2008). Likewise, methanogens acquire their energy from waste products such as hydrogen molecules that are produced by other obligate anaerobes (Dridi et al., 2011). Conversely, the gut microbiota adapted a negative antagonistic relationship with pathogens/pathobionts to suppress their growth either directly *via* the production of bacteriostatic/bactericidal substances against pathogens or indirectly where commensal bacteria communicate with the host *via* their cell surface antigenic molecules such as lipopolysaccharides (LPS) and peptidoglycans (PGNs) and produce metabolites which either promote the host gut barrier physiology to restrain pathogen/gut microbiota translocation into the systemic circulation or activate the host mucosal/systemic immunity to prevent pathogen colonization in the gut. Commensal bacteria also adapted several other ways to restrain pathogen colonization, such as competing for nutrients, occupying a specific niche, and changing the gut physiological environment (Kamada et al., 2013; Sassone-Corsi and Raffatellu, 2015; Ducarmon et al., 2019; Pickard and Núñez, 2019). On the other hand, pathogenic microbes or pathobionts also evolve direct or indirect strategies like commensals to overcome commensal-mediated colonization resistance and expand their growth to cause infection, which underlies the discussion of microbe–microbe and microbe–host interaction (Baümler and Sperandio, 2016; Rolhion and Chassaing, 2016).

Consequently, the gut microbiota community interactions in the intestine can be categorized into three major themes, namely, microbial–host, microbial–environmental, and microbial–microbial interactions, that dictate the distribution of individual microbial species membership and abundance across the GIT, which may lead to interindividual gut microbiota differences in composition and density as well as to variable susceptibility to diseases between individuals (Pridmore et al., 2004; Ley et al., 2006; Turnbaugh et al., 2008; The Human Microbiome Project Consortium, 2012). Various previous studies reported the relevance of the gut microbiota in host health outcomes and their disruption with multiple chronic metabolic and inflammatory diseases (Benítez-Páez et al., 2020; Lapidot et al., 2020; Parhi et al., 2020; Ryan et al., 2020), where microbial–host, microbial–environmental, and/or microbial–microbial interactions were altered, but limited work is available on the gut microbiota in the relevance of resistance to pathogen colonization and its disruption impact on pathogenic infections.

**Figure 1:**
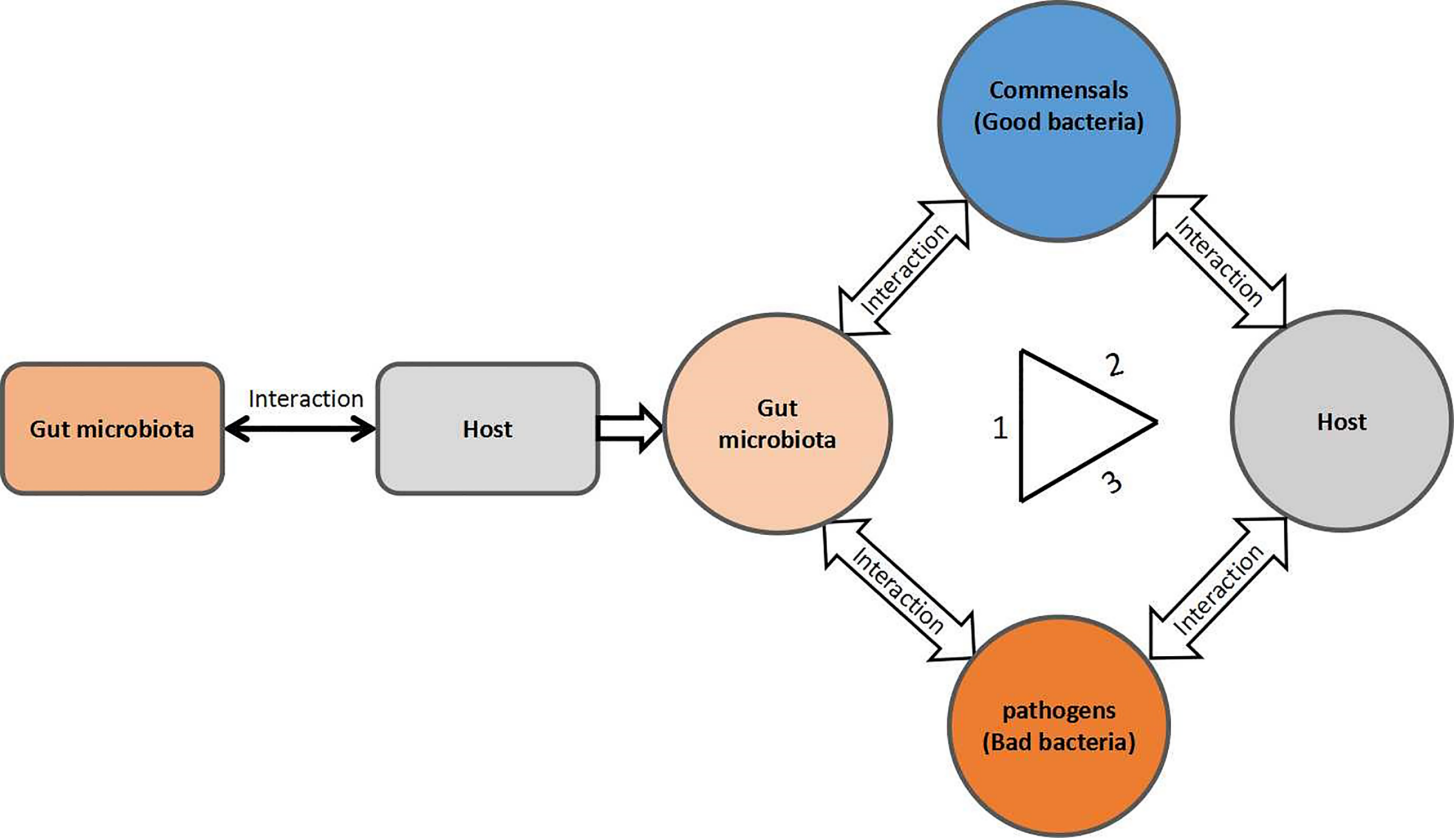
A triangular form of the gut microbial community interactions. 1) Commensal–pathogen interactions; 2) commensal–host interactions; and 3) pathogen–host interactions.

The current review article focuses on; gut microbiota interaction; microbe–microbe; microbe–host interactions in pathogen colonization resistance and infection prevention. The gut microbiota interactions within the community and host form a triangular network, as summarized in **Figure 1**. Therefore, any disruption in the typical regular gut microbiota composition may interrupt this triangular network of the gut microbe–microbe interaction within the community and microbe–host interaction, yielding a bloom in pathogenic/pathobiont bacteria population and their associated infections. In this review, we make an effort to review the available literature on the perturbation of the gut microbial community from the perspective of gut microbial colonization resistance and pathogenic bacteria-associated infectious diseases and their underlying mechanism.

As the majority of the mechanistic studies have been conducted in *S. Typhimurium*, enterohaemorrhagic *Escherichia coli* (EHEC), and *C. difficile* to investigate these interactions; therefore, this review covers these pathogenic organisms more extensively than others.

## Diet and Gut Microbiota Composition

Among the gut microbiota regulating factors, diet is one of the major determinants that define the composition and distribution of the microbiota in the different compartments of the GIT. The mammalian host is colonized with a simple structured gut microbiota immediately after birth; however, with age, as the diet changes from breastfeeding to fiber-rich nutrition, the gut microbial structure and composition is altered dramatically (Hasegawa et al., 2010; Matamoros et al., 2013). The simple sugar molecules and amino acids are rich in the small intestine, readily absorbed by the host cells; thus, carbon as an energy source is not or less available for microbes to use in that region. In contrast, the complex polysaccharide molecules derived from plant or animal sources are indigestible by the host and move to the colon. So, energy sources for bacterial growth are substantially altered across the GIT compartment. As a result, the gut microbiota composition and density are also variable along with the lower GIT from the upper small intestine down to the lower end of the colon. The upper part of the colon is populated with a high density of Proteobacteria and Lactobacillales, and their number is reduced in the large intestine colon, implying that Proteobacteria, such as *Escherichia coli*, cannot digest complex carbohydrates and cannot use them as energy sources. In contrast, the colon is colonized with a high density of Bacteroides and Clostridiales, implying that these bacteria may have hydrolytic digestive enzymes and can use the complex polysaccharide polymers as an energy source. Consequently, the abundance of Proteobacteria and Lactobacillales is much lower in the colon, whereas Bacteroides and Clostridiales are the dominant populations in the large intestine (Koropatkin et al., 2012). The given literature suggests that diet content can significantly influence the relative abundance of microbial taxa and their distribution in the gut. Thus, nutrient content works as a major driving force in defining the microbial community structure in the intestine (Koropatkin et al., 2012). In addition, it also indicates that usually in fiber-rich diet consumption, the density of the beneficial bacterial is dominant over pathobiont or pathogen counterparts such as *E. coli*. Furthermore, diet has been acknowledged for its profound effect on the gut microbiota composition to host physiology, immunity, and susceptibility to infectious diseases (Kau et al., 2011). Dietary choices have successfully affected the susceptibility to enterohemorrhagic *E. coli* (EHEC) serotype O157:H7 (*E. coli* O157:H7) infection in mice, where the high fiber diet (HFD)-fed mice had shown higher *E. coli* O157:H7 colonization level and more severe infection than in mice fed with the low fiber diet (LFD) (Zumbrun et al., 2013). The administration of diet with phytonutrient supplementation expanded the growth of beneficial bacterial Clostridia species that protect mice colonization by the pathogen *Citrobacter rodentium* (Wlodarska et al., 2015) [*C. rodentium*, a mouse bacterium that is used extensively in mouse models as a surrogate for the human enteric pathogens EHEC and EPEC (enteropathogenic *E. coli*)] (Schauer and Falkow, 1993; Law et al., 2013). However, the discrepancy in microbial diversity and its distribution among individuals is multifactorial and cannot be described by a single factor alone. For example, *Bifidobacteria*, a commensal bacterium, abundantly colonizes the human intestine, affecting its response to pathogen attack (Fukuda et al., 2011). Germ-free or antibiotic-treated mice challenged with pathogenic species showed severe enteric infection than wild-type mice, indicating that the interaction of gut-resident microbes and pathogens may affect disease outcomes (Sprinz et al., 1961; Zachar and Savage, 1979; Ferreira et al., 2011; Kamada et al., 2012). This explanation implies that the gut microbiota diversity and density distribution may also be affected by microbe–microbe and microbe–host interactions. Therefore, additional ecological analyses of intracommensal interactions and better characterization of the metabolic activities of individual bacteria are required to completely understand the microbial ecosystem in the intestine.

## Pathogen Resistance by Commensal Gut Microbiota

The theme is that gut microbiota has an effect on the risk and course of the host enteric diseases either by resistance or assistance to the colonization of the host by pathogenic microbial species. Several preclinical animal modeling studies have shown that the microbiota can promote resistance to colonization by pathogenic species (Bohnhoff et al., 1954; Cameron and Sperandio, 2015; Pacheco and Sperandio, 2015; Sassone-Corsi and Raffatellu, 2015). The germ-free and antibiotic-treated mice experienced a more severe enteric infection and showed high susceptibility to enteric pathogens such as *S.* Typhimurium, *Shigella flexneri*, *Listeria monocytogenes*, and *C. rodentium*, than conventionalized wild-type or specific pathogen-free (SPF) mice (Sprinz et al., 1961; Zachar and Savage, 1979; Ferreira et al., 2011; Kamada et al., 2012). Similarly, some microbiota has led to the expansion or enhanced the virulence of the pathogenic microbial population and results in severe infection (Cameron and Sperandio, 2015). The impact of the gut microbiota on pathogen colonization resistance is best explained by a microbial transfer experiment, where transplantation of microbiota from a strain of mice infected with *C. rodentium* induced a similar susceptibility in mice that were resistant before, and the transplantation of microbiota from an insusceptible animal led to resistance against pathogen infection in animals which were highly susceptible before (Ghosh et al., 2011; Willing et al., 2011). In addition, the concern is how the differences in the gut microbiota composition affect susceptibility to pathogen infection. A human clinical survey study further reinforces this idea. For example, a Swedish study reported that susceptibility to *Campylobacter jejuni* infection was shown to be dependent on the gut microbiota species composition. Individuals with higher diversity and richness of microbiota and with a high count of bacterial species from the genera *Dorea* and *Coprococcus* showed significant resistance to *C. jejuni* infection. By contrast, those individuals who had a lower microbial diversity and with a low count of bacterial species from the genera *Dorea* and *Coprococcus* showed high susceptibility to infection with *C. jejuni* (Kampmann et al., 2016). The treatment of pathogen infection in animal models with gut microbiota transfer from a healthy donor, with probiotic intervention, or with microbial metabolite administration further validates the gut microbiota relevance to pathogen colonization resistance and prevention of pathogen-associated infections (Hsiao et al., 2014; Fan et al., 2015; Steed et al., 2017; Jacobson et al., 2018; Alavi et al., 2020; Burgess et al., 2020; Kim et al., 2020; Winkler et al., 2020).

## Gut Microbiota and Pathogen Colonization Resistance Mechanisms

### Commensals Regulate Pathogen Growth and Activity

**Figure 2:**
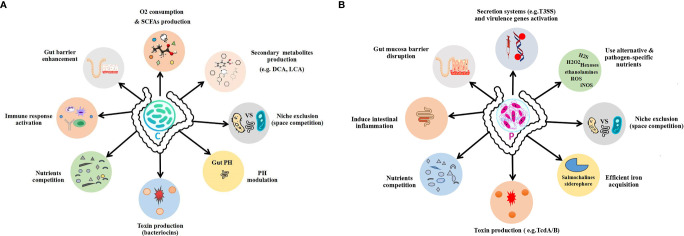
Outline of the gut commensal and pathogen mechanisms. **(A)** Commensal colonization resistance mechanisms. **(B)** Pathogen expansion mechanisms to overcome commensals. C, commensal; P, pathogen.

**Figure 3:**
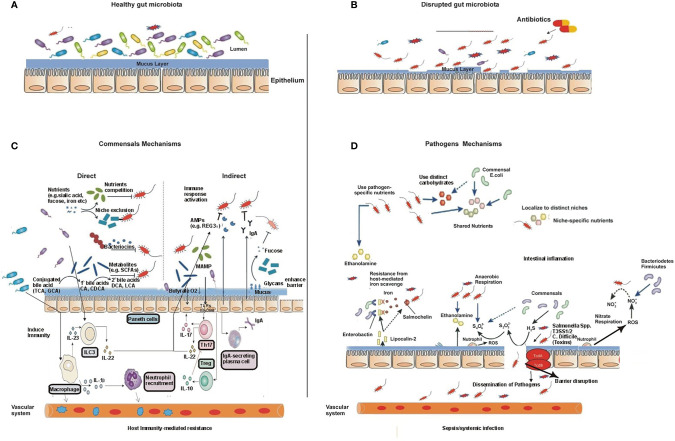
The gut microbiota during health and disease and their mechanisms. **(A)** A diverse and non-disturbed microbiota confers resistance to colonization by enteric pathogens in the intestinal epithelium. **(B)** Treatment with antibiotics decreases the diversity of the microbiota and leads to the expansion of the pathogen population. **(C)** The mechanisms of intestinal microbiota-mediated colonization resistance: in the healthy state, the resident commensal bacteria occupy the entire intestinal colonization niches and mediate colonization resistance through several direct and indirect mechanisms, thereby suppressing the proliferation and colonization by exogenous enteric pathogens and resident opportunistic pathobionts. Examples of gut microbiota-mediated direct inhibition of pathogens from intestinal colonization include the following: 1) competition for nutrients and production of toxic substances such as bacteriocin, secondary bile acids, and fermentation products such as short-chain fatty acids; these microbiota-derived products directly inhibit the growth of pathogens and pathobionts. 2) Commensals can also modify virulence factor expression in pathogens by consuming residual oxygen or suppressing growth by their metabolites. Specific commensals reduce pathogen adherence to the intestinal mucosa due to having high diversity or possessing unique adhesion molecules, and the process is termed as niche or adhesion exclusion. Similarly, gut commensals mediate colonization resistance *via* a variety of indirect means. 3) Gut microbiota enhances the gut barrier function through upregulation of the mucus through the release of antimicrobial peptides, such as Regγ, and regulating IgA secretion. Similarly, microbiota activates host immune response and provides colonization resistance. Gut microbiota stimulates the priming of intestinal macrophages through IL-1β, which promotes the recruitment of neutrophils to the site of infection and eradicates pathogens. Commensal microbiota promotes differentiation and/or activation of Th17 cells and innate lymphoid cells (ILCs), which control both commensals and pathogens through secreted cytokines, such as IL-22- and IL-22-dependent antimicrobial peptides. Thus, commensals boost both innate and adaptive mucosa immunity and prevent pathogen colonization. Disruptions of the commensal gut microbial community by antibiotics or other environmental incursions result in increased colonization by pathogens. As a result, pathogens may potentially disseminate systemically and induce septic shock and/or systemic organ infection. **(D)** The mechanisms of pathogens to overcome commensal-mediated resistance: pathogens resist commensals through multiple strategies. 1) Pathogenic bacteria/enteric pathogens overcome commensals *via* specific alternate nutrients such as carbohydrates and ethanolamine. 2) Pathogens adhere to a pathogenic specific niche on the intestinal epithelial surface that is devoid of commensal microbiota through the expression of adhesion molecules, such as intimin. 3) Pathogens induce intestinal inflammation, which alters the gut nutritional and physiological environment and inhibits the growth of commensal bacteria, thus conferring an advantage to enteric pathogens. Pathogens trigger intestinal inflammation and use its virulent factors such as T3SS-1 and T3SS-2 (*S.* Typhimurium) or toxins (*C. difficile*), which results in the release of antimicrobial molecules such as reactive oxygen species (ROS) and reactive nitrogen species (RNS), e.g., inducible nitric oxide synthase (iNOS), from host epithelial cells, converted into nitrate (NO^3−^), which can be utilized by pathogenic bacteria as an energy source through nitrate respiration, while commensals lack this ability, thus having a growth advantage over commensals. Similarly, a high influx of neutrophils during inflammation to the site of infection produces ROS, which enable the conversion of $S_{2}O_{3}^{2-},$ generated by commensal bacteria, into $S_{4}O_{6}^{2-},$ which can be used by pathogens through anaerobic respiration but cannot be used by commensals as an electron acceptor to extract energy that further boosts the growth of pathogenic bacteria such as *S.* Typhimurium. Similarly, lipocalin-2 is an anti-siderophore molecule, produced by the host cell during pathogen infection, which prohibits iron uptake by commensal bacteria by binding to the bacterial siderophore, enterobactin, which can block the growth of commensals such as Enterobacteriaceae that rely on the siderophore enterobactin for the acquisition of iron (Fe^3+^). However, pathogens such as *Salmonella* spp. have a distinct siderophore, salmochelin, for iron uptake, so it does not bind to the *S.* Typhimurium siderophore salmochelin, which is resistant to lipocalin-2-mediated inhibition.

In the gut microbiota, both the commensals and pathogens require a common source of energy, habitat, and nutrients, for which they must struggle to best adapt in the intestine of the host, to colonize and grow. Therefore, they must evolve certain mechanisms to best utilize these resources and outcompete each other. Generally, commensal bacteria regulate the population and activity of the pathogenic bacteria either by direct or indirect ways to maintain the normal healthy gut microbial composition while using the axes of microbe–microbe and microbe–host interactions, shown in **Figure 1**. In the direct mechanism to prohibit pathogen colonization by commensals, the commensals mediate colonization resistance by killing the pathogens or reducing their growth by producing toxic chemical substances such as bacteriocins, secondary bile acids, and proteinaceous toxins (Schamberger and Diez-Gonzalez, 2002; Hammami et al., 2013; Ducarmon et al., 2019; Pickard and Núñez, 2019), changing the gut physiological environment (pH alteration) (Cherrington et al., 1991; Shin et al., 2002; Fukuda et al., 2011) and nutrient competition (Momose et al., 2008a; Momose et al., 2008b; Fabich et al., 2008) as well as through specific metabolite production (Gantois et al., 2006; Pacheco et al., 2012). On the other hand, in the indirect mechanism, the commensals in the gut microbiota combat the pathogens mainly *via* induction of the host immune response against pathogens (Satoh-Takayama et al., 2008; Vaishnava et al., 2008; Zheng et al., 2008; Ivanov et al., 2009). The overall mechanisms of commensal colonization resistance are shown in **Figures 2** and **3**.

#### pH Alteration and Pathogen Growth

Optimum pH is highly critical for the growth of most enteropathogenic bacterial species such as *Bacillus cereus*, *E. coli*, and enterotoxigenic bacteria (Ceuppens et al., 2012; Hammami et al., 2013). To downregulate the pathogenic bacterial growth, the commensal bacteria evolve certain strategies that alter the gut local physiological environment by modulating the pH and that prevent pathogen colonization and reduce the risk of intestinal infectious diseases (Gantois et al., 2006; Turovskiy et al., 2011). Certain commensal bacteria produce short-chain fatty acids (SCFAs), i.e., butyric acid, propionic acid, and acetic acid, major metabolic products of most gut microbial fermentation, which modulates the gut pH and prevents the proliferation of certain intestinal pathogenic microbe populations (Cherrington et al., 1991; Shin et al., 2002). In a mouse model study of enteric pathogen *S.* Typhimurium infection, the Bacteroides species showed resistance against *S.* Typhimurium colonization by changing the pH of the gut through the production of propionate that limits its growth (Brinkman et al., 2013). The probiotic species *Bifidobacterium* has blocked the pathogenic *E. coli* growth by reducing its environment pH (Fukuda et al., 2011). In addition, commensals also produce certain metabolites that can directly inhibit specific microbial members of the same or related bacterial species. For example, bacteriocins from *E. coli* directly inhibit the growth of related pathogen EHEC (Schamberger and Diez-Gonzalez, 2002; Hammami et al., 2013). Although commensal bacteria resist pathogen colonization and reduce the risk of pathogens associated with intestinal infections *via* modulating the gut pH, however the underlying molecular mechanism of the phenomenon is partially or completely unexplored.

##### Nutrient Competition, Metabolite Production, and Pathogen Activity

The preferential consumption of nutrients by commensals, which are required by pathogenic bacteria for their growth, is an alternative strategy of the commensal bacterial community to outcompete the pathogenic microbes. For example, commensal *E. coli* with EHEC competes for amino acids, organic acids, and other nutrients (Momose et al., 2008a; Momose et al., 2008b; Fabich et al., 2008; Leatham et al., 2009). By consuming the commonly available limited nutrient resources, the commensal community causes the starvation of the competing pathogenic bacteria. Commensal bacteria also produce certain toxic chemicals which affect the pathogen virulence and compromise their proliferation. For example, butyrate, a short-chain fatty acid, downregulates the expression of several virulence genes, including those of the secretion system type 3 proteins (SST3) in the enteropathogenic species, i.e., *Salmonella* Enteritidis and *S.* Typhimurium (Gantois et al., 2006). Similarly, fucose, a host mucin-derived component, is generated as a fermentation product by a commensal bacterium *Bacteroides thetaiotaomicron*, which possesses fucosidase activity that affects the expression of the virulent factor Ler that works as a key regulator of the locus of enterocyte effacement (LEE) genes in EHEC (Pacheco et al., 2012). Thus, commensals not only modulate and affect the pathogen virulence directly *via* the production of metabolites but also suppress the pathogen virulence genes by altering the physiological condition required for the virulence activity of the pathogens. For example, high oxygen tension is required for the virulent gene expression of *S. flexneri* to produce the Shiga toxin. In response, the commensal facultative anaerobic bacteria such as *E. coli*, a member of the Enterobacteriaceae family, consume the available residual oxygen, leading to the partial expression of the *S. flexneri* virulent factors in the gut lumen (Marteyn et al., 2010).

##### Commensals Control Pathogens by Stimulating the Host Immunity

Gut commensals also mediate pathogen colonization resistance to prevent pathogen infection indirectly by enhancing gut immunity, including promoting the functionality of the gut barriers and innate immune responses. The epithelium layer/barrier is the first line of defense against any invading pathogen. The concept that gut epithelial barriers are functionally regulated by the gut microbiota is primarily supported by indirect evidence. For example, the germ-free and mice deficient with proteins involved in microbial recognition, such as NOD2 and MYD88, had shown with impaired production of antimicrobial peptides, particularly by Paneth cells of the small intestine (Kobayashi et al., 2005; Vaishnava et al., 2008). MYD88-deficient or Paneth cell-deficient mice have shown abnormal gut barrier function and a high level of the pathogenic bacterial load inside the gut mucosa (bacterial translocation) due to their inability to produce sufficient and specific antimicrobial peptides to prevent pathogen colonization (Vaishnava et al., 2008). In addition, antimicrobial peptides not only prevent pathogen infections by direct killing but also limit pathogen bacteria colonization. For example, mice deficient in MYD88 adaptor protein or Paneth cell have shown higher mucosa-associated bacterial load in the small intestine than wild-type littermates (Vaishnava et al., 2011).

Additionally, the intestinal microbiota not only enhances the gut barrier function but also promotes host innate immunity to resist enteric pathogen infection. IL-1β is a cytokine typically produced during active infection that is critical for enteric pathogen elimination. The gut microbiota has been recognized with homeostatic level production of pro-IL-1β in the intestine-resident macrophages. The production of pro-IL-1β from the resident gut macrophages is MYD88 dependent, which is regulated by the gut microbiota. Thus, the gut microbiota regulates the priming of the macrophages to rapidly respond to the invading pathogens by converting pro-IL-1β into mature active IL-1β to prevent its colonization (Franchi et al., 2012). Gut microbiota can also induce host immunity *via* an MYD88-independent manner. For example, colonization of germ-free mice with commensal bacteria produces the T helper 17 (Th17) cell in the intestinal tissue, which confers resistance to enteric pathogen infection, such as *C. rodentium*, which is independent of microbial recognition signaling molecules such as MYD88, TRIF, and RIP2 (Ivanov et al., 2009). Interleukin 17-producing Th17 cells and a certain subset of dendritic cells are a group of type 3 innate lymphoid cells (ILC3), producing interleukin 22 (IL-22), which is involved in the upregulation of the gut barrier protein REGIIIγ (Satoh-Takayama et al., 2008; Zheng et al., 2008; Sanos et al., 2009). IL-22-mediated production of REGIIIγ by the intestinal epithelium is protective against enteric infection by *C. rodentium* (Satoh-Takayama et al., 2008; Kiss et al., 2011; Qiu et al., 2012). The role of the commensal bacteria for the production of REGIIIγ and elimination of pathogenic bacteria has been more specifically justified by the administration of the bacterial-associated antigenic molecule lipopolysaccharide (LPS) or bacterial flagellin that upregulated REGIIIγ and enhanced the eradication of *Enterococcus* species, known as vancomycin-resistant *Enterococcus* (VRE) (Brandl et al., 2008; Kinnebrew et al., 2010). In addition to the production of antimicrobial peptides, interleukins, the gut microbiota signaling is likely through MYD88 which may also enhance the gut barrier function *via* secretory antibody immunoglobulin A (IgA) production from intestinal epithelial cells. Secretory IgA mediates pathogen resistance by binding to specific microbial antigens and neutralizes pathogen activity, thus preventing pathogen colonization to cause infection (Fagarasan et al., 2010; Suzuki et al., 2010; Strugnell and Wijburg, 2010; Frantz et al., 2012). In host mucosal immunity, antimicrobial peptides and IgA are also involved in gut microbiota shaping; however, the composition of the gut microbiota is regulated by multiple factors; therefore, its remain unclear that these two factors alone are the main determinants for pathogen resistance (Petnicki-Ocwieja et al., 2009; Salzman et al., 2010; Vaishnava et al., 2011; Macpherson et al., 2012).

Furthermore, the role of commensals taking advantage of the host immune response and mediating pathogen colonization resistance is clearly visible from studying pathogen infections in animal models. For example, a mouse model of sepsis induced with human sepsis-associated bacterial pathogen was rescued with fecal microbiota transplant (FMT) by enhancing pathogen clearance *via* restoration of the host systemic immunity. This effect of FMT is linked to the upregulation of butyrate-producing Bacteroidetes, which increased the expression of interferon (IFN) regulatory factor 3 and alleviated the disease pathology. The study suggested that FMT may be a possible therapeutic option in sepsis, related to the host immunosuppression (Kim et al., 2020). A study on a mouse model of parasitic protozoa reported that colonization of *Clostridium scindens* protects from *Entamoeba histolytica* colitis, where the gut microbiota species cross-talk with bone marrow and regulate susceptibility to amebiasis *via* innate immunity activation. The protection mechanism of the intestinal bacterium *C. scindens* against *E. histolytica* is dependent on bile salt metabolism. The bile salt-derived metabolite deoxycholate activates the host bone marrow epigenetically and increases the immune response by inducing the immune cells granulocyte monocyte progenitors (GMPs) and neutrophils into the gut that prevent the colonization of *E. histolytica* (Burgess et al., 2020). The gut microbiome was also found to protect against virus infection in a mouse model by modulating the host innate immunity. The intestinal bacterium *C. scindens* has restricted the alphavirus (CHIKV) infection and dissemination in a mouse model by restoring the antiviral immune response type I IFN signaling through its associated metabolite deoxycholic acid production (Winkler et al., 2020). Similarly, *Clostridium orbiscindens*, a human intestinal bacterium, and its derived metabolite desaminotyrosine (DAT), has rescued mice infected with influenza virus through modulation of type I IFN signaling (Steed et al., 2017). The probiotic *Bacillus* bacterial species have been found to eliminate *Staphylococcus aureus* infection by inhibiting/reducing its intestinal colonization through signaling interference (quorum sensing) of *S. aureus*. Quorum sensing is a signaling mechanism through which bacteria manage their gene expression and metabolism according to their population density (Piewngam et al., 2018). *Candida albicans* colonization in the GIT has been restricted by commensal anaerobic bacteria—specifically Firmicutes (clostridial clusters IV and XIVa) and Bacteroidetes—through induction of HIF-1α and LL-37 that activated innate immunity effectors in the gut (Fan et al., 2015). *Corynebacterium mastitidis*, an ocular commensal, protects the cornea from pathogenic *C. albicans* and *Pseudomonas aeruginosa* infection by inducing the IL-17 production from resident mucosal gd T cells (Leger et al., 2017). The gut microbiota is also recognized in the protection from malaria parasite transmission by eliciting a specific immune response (Yilmaz et al., 2014). In addition, the gut microbiota mediates systemic immune effects *via* immunoglobulin G (IgG) production that safeguard against systemic infections by *S.* Typhimurium and *E. coli* (Zeng et al., 2016). *Enterococcus faecium*, a human commensal bacterium, protects against *S.* Typhimurium infection in mice *via* the production of antimicrobial peptides through an associated unique peptidoglycan hydrolase secreted antigen A (SagA). SagA interacts with the pattern recognition receptors found in the host gut epithelial cells and elicits an innate immune response against the *Salmonella* pathogen (Pedicord et al., 2016). This brief discussion justifies that commensal bacteria restrain pathogen growth using the host immunity *via* a variety of mechanisms.

## Disruption of the Gut Microbiota and Pathogen Outgrowth

Disruption of the gut microbiota, most commonly associated with antibiotic usage, has been known for its rapid, dramatic, and sometimes everlasting effect on the gut microbiota composition and activity and can alter the microbial taxonomic and functional profile (Modi et al., 2014). It decreases the diversity of commensal bacteria, which compromises resistance to colonization by incoming invading pathogenic bacteria or by existing pathobiont expansion (Modi et al., 2014). The loss of commensal-mediated colonization resistance against pathogens by antibiotics increases the individual susceptibility to enteric pathogen infections such as by *S.* Typhimurium and EHEC and most notably leads to substantial growth in the abundance of *C. difficile*, followed by a severe intestinal inflammation (Rupnik et al., 2009; Ayres et al., 2012; Modi et al., 2014; Grünewald and Ruf, 2016; Mullineaux-Sanders et al., 2018). *S.* Typhimurium, EHEC, and *C. difficile* are typically the pathogenic bacterial species used for most mechanistic studies investigating such interactions (Modi et al., 2014; Grünewald and Ruf, 2016). *C. difficile* is a leading nosocomial infectious disease associated with diarrhea and colitis (Rupnik et al., 2009). Typically in the intestine of a healthy human, *C. difficile* growth is suppressed by commensals, thus controlling its presence and number; however, a substantial increase was seen after treatment with broad-spectrum antibiotics in hospitalized patients, followed by an acute intestinal inflammation (Ferreyra et al., 2014b; Rupnik et al., 2009; Grünewald and Ruf, 2016). Like in human, the mouse *C. difficile* infection model has also reported that *C. difficile* could not colonize and induce intestinal inflammation in wild-type mice, whereas antibiotic therapy enhanced the incidence of *C. difficile* infection, which did not disseminate systemically but caused gross damage to the intestinal epithelial barrier *via* production of their associated toxins TcdA and TcdB (Rupnik et al., 2009; Ng et al., 2010). In consequence, the toxin-mediated epithelial barrier disruption has led to the systemic dissemination of the gut microbial species, which may induce lethal septic shock (Hasegawa et al., 2012). Thus, for *C. difficile* to induce colitis, the use of antibiotics is one of the most important risk factors (Grünewald and Ruf, 2016). Similarly, *S.* Typhimurium is another bacterial enteric pathogen that is commonly associated with food ingestion and poorly colonizes the mouse gut during the normal physiological condition due to the presence of the resident commensal microbial community (Bohnhoff et al., 1954). However, when the resident gut microbial community is disrupted with antibiotic use, or the recipient mice have low complexity and reduced diversity of the gut microbiota, the pathogen can freely proliferate and induce inflammation (Bohnhoff et al., 1954; Pavia et al., 1990; Endt et al., 2010). In addition, an altered gut microbial community structure may also facilitate the proliferation and outgrowth of the potentially harmful pathobionts of the intestinal indigenous bacteria. For example, *E. coli* is an avirulent bacteria; normally, its growth is suppressed by the gut commensal microbes; however, its overgrowth and accumulation occur with antibiotic use and can disseminate across the gut mucosa systemically following intestinal epithelial injury by a stimulus such as dextran sulfate sodium (DSS), thereby inducing intestinal inflammation in mouse (Ayres et al., 2012). Furthermore, VRE causing sepsis in immunocompromised individuals has been associated with antibiotic treatment (Arias and Murray, 2012).

Normally, commensal gut bacteria regulate the harmful subset of bacteria, including VRE, through induction of innate immunity such as the production of antimicrobial peptides, for example, REGIIIγ, particularly more important for bacterial killing, which has been found with the eradication of VRE (Brandl et al., 2008; Kinnebrew et al., 2010). However, a recent study suggested that a specific intestinal bacterial consortium facilitates the clearance of the VRE species, which works independently of the host innate immune pathways, such as MYD88 signaling (Ubeda et al., 2013). Although the specific mechanism is unknown, it may be related to the direct mechanism such as competition for the common limited source of nutrition or the commensal gut microbiota mediates VRE eradication *via* the indirect mechanism (immune response induction), or both mechanisms depend on different regions of the intestine. For example, in the small intestine, the production of antimicrobial proteins might be a primary mechanism for clearance of VRE, whereas direct killing/inhibition by a specific microbial population may be a primary mechanism in the colon (Ubeda et al., 2013).

## Strategies of Pathogens to Overcome Commensal Resistance and Cause Infection

As described earlier, multiple strategies have been developed by the commensal gut microbiota that mediates pathogen colonization resistance to prevent pathogen infection. Similarly, on the other hand, pathogens also evolved counter mechanisms to escape from these regulatory mechanisms and dominate over commensals and cause infection. For instance, to counteract nutrient competition by commensals, certain pathogens have developed strategies to use alternative nutrients or to utilize the commonly available nutritional resources more efficiently (see **Figures 2**, **3**).

### Nutrient Competition

In the human gut, the simple sugars are absorbed in the small intestine, and the complex polysaccharides and host glycan are available as energy-rich sources in the colon; therefore, the most abundant microbiota are those that are able to use these undigested complex polymers as a nutrient source in the colon (Ferreyra et al., 2014a). The gut epithelium mucosal layers, which are known as mucin, act as protective barriers, rich in sugar components, such as sialic acid, fucose, galactose, *N*-acetylglucosamine, *N*-acetylgalactosamine, and mannose. These sugar molecules are metabolized by saccharolytic members of the gut bacterial community, such as members of the Bacteroidales, making them available as an energy source for those members that are unable to harvest these sugars, and the pathogenic bacteria can also utilize these available nutrients for the proliferation of their growth (Rakoff-Nahoum et al., 2014). Several previous studies have investigated these syntrophic interactions in the gut microbiota members of Bacteriodales, where *B. thetaiotaomicron* is used as a model species. *B. thetaiotaomicron* harbors multiple hydrolytic enzymes and has the ability to catabolize host glycan multiple components (Alverdy et al., 1985; Bourlioux et al., 2003; Chow and Lee, 2008; Fischbach and Sonnenburg, 2011; Ng et al., 2013). For example, *B. thetaiotaomicron* has sialidase activity to release the sugar component sialic acid from the gut epithelial mucins but lacks the capability to utilize it; however, the bacterium gains access to the underneath glucans to use it as an energy source while releasing sialic acid. The release of sialic acid by the bacterium *B. thetaiotaomicron* enhances its availability in the colon, which can be used by pathogenic bacteria such as *C. difficile* and *S.* Typhimurium as an energy source, which provides them a growth advantage over commensals (Ng et al., 2013). Therefore, microbiota and pathogenic bacteria that use sialic acid as their energy source depend on the presence and activity of *B. thetaiotaomicron*, as reported that *B. thetaiotaomicron* mutants that have no sialidase activity, are failed to give a growth advantage to these two pathogenic bacteria i.e. *C. difficile* and *S.* Typhimurium (Ng et al., 2013). Similarly, *B. thetaiotaomicron* also releases sugar fucose from the epithelial mucus layer, results in an increase in the availability of this sugar in the gut lumen (Alverdy et al., 1985; Bourlioux et al., 2003; Chow and Lee, 2008; Fischbach and Sonnenburg, 2011), which can be used as an energy source by *S.* Typhimurium (Ng et al., 2013). More importantly, *B. thetaiotaomicron* has shown enhancement in the fucosylation of the mucosal glycan in monoassociated germ-free mice (Bry et al., 1996; Hooper et al., 1999). The gut-resident microbiotas almost occupy the entire lumen or adhere to the outer mucus layer of the intestinal epithelium, and the pathogenic bacteria such as EHEC compete to achieve a unique niche by adhering to the gut epithelial enterocytes; therefore, the pathogenic bacterium EHEC must struggle for nutrients to successfully outcompete the commensal microbiota. EHEC has the ability to colonize the intestine due to the freely available simple sugar molecules that can be used by the commensal *E. coli* as well. Commensal *E. coli* has multiple strains with shared nutritional requirements with EHEC; hence, the intestinal colonization of the mouse by EHEC can interfere with *E. coli* (Maltby et al., 2013). A mouse model of EHEC treated with streptomycin while having three distinct strains of commensal *E. coli* was used to examine the differential sugar requirements for the successful intestinal colonization of the mouse. EHEC has successfully colonized in the mouse model that was only precolonized with at least one commensal *E. coli* strain but failed to colonize in mice that were precolonized with all three commensal *E. coli* strains (Maltby et al., 2013). In normal circumstances, EHEC can only utilize monosaccharides and disaccharides, which can also be used by the commensal bacteria *E. coli*; thus, commensal *E. coli* is the only main competitor of EHEC that utilizes the simple sugar fucose as a preferential source of carbon in the mammalian intestine (Fabich et al., 2008; Kamada et al., 2012). To counteract and compete *E. coli*, EHEC has employed certain catabolic pathways to metabolize several distinct alternative sources of sugar simultaneously, such as hexuronate, glucuronate, galacturonate, and sucrose during colonization of the gut, which is not employed by the gut commensal *E. coli* (Fabich et al., 2008; Maltby et al., 2013), thus resulting in the expansion of EHEC growth (Autieri et al., 2007; Fabich et al., 2008; Bouguénec and Schouler, 2011). The loss of this polymetabolic capability has an additive effect on intestinal colonization of EHEC, whereas this event is not observed in commensal *E. coli*, which predicts that *E. coli* utilizes freely available sugar molecules in a stepwise manner (Fabich et al., 2008). Therefore, EHEC and commensal *E. coli* have differences in the colonization of the mammalian intestine due to their difference in metabolic strategy for energy extraction and the use of nutrients. In contrast, *B. thetaiotaomicron* has a diverse source of nutrients and does not need to compete with EHEC such as *C. rodentium*, because it can use polysaccharides. So, when a diet contains both monosaccharides and polysaccharides, then *B. thetaiotaomicron* prefers to use polysaccharides instead of competing for monosaccharides, and as a result, *C. rodentium* is not cleared by *B. thetaiotaomicron*. However, *B. thetaiotaomicron* is forced to compete with *C. rodentium* for monosaccharides and clears it from the mouse intestine in case when a diet contains only monosaccharides (Kamada et al., 2012). In addition, another study reported that although *C. rodentium* exhibits a similar nutritional and metabolic profile as the non-pathogenic commensal *E. coli* (Kamada et al., 2012), however, it resides in a unique niche on the intestinal epithelium surface, where commensals cannot reside normally, because *C. rodentium* expresses a distinct adhesion molecule known as intimin, encoded by LEE genes; therefore, *C. rodentium* lives in a different environmental region and has no need to compete for nutrients with commensals (Kamada et al., 2012).

Moreover, ethanolamine is an abundant source of carbon and of nitrogen release as a by-product into the intestine lumen during the intestinal epithelial cell turnover (Bertin et al., 2011) that can be used by several pathogenic bacterial species as a pathogen-specific nutrient (Garsin, 2010), while it cannot be used by the majority of the gut commensal bacteria (Korbel et al., 2005). Pathogenic bacteria, particularly of food origin, are specially adapted to use it, such as by EHEC, due to having the *eut* operon in its genome for ethanolamine metabolism (Perna et al., 2001; Fabich et al., 2008; Bertin et al., 2011). In contrast, non-pathogenic commensal *E. coli* do not possess the *eut* operon and cannot utilize ethanolamine as a nutrient (Korbel et al., 2005; Bertin et al., 2011). Consequently, the bacteria *S.* Typhimurium, EHEC, and *L. monocytogenes* have favorable growth over the commensals in the intestine due to their capability to metabolize ethanolamine as an energy source (Joseph et al., 2006; Bertin et al., 2011; Thiennimitr et al., 2011). Moreover, some pathogenic bacteria use effective mechanisms for nutrient uptake and consume the available common sources of energy more efficiently than commensals. For example, many bacteria produce siderophore, an iron-chelating small molecule, to acquire iron in sufficient quantity, which is an essential component for bacterial growth (Crosa and Walsh, 2002). In response, lipocalin-2 (Lcn2) is produced by host cells that block the 2,3-dihydroxy benzoate-based siderophore enterobactin (Ent) in the commensal *E. coli*, thereby stopping the iron acquisition and growth proliferation of the commensal *E. coli*. In contrast, pathogenic bacteria such as pathogenic *E. coli*, *S.* Typhimurium, and *Klebsiella pneumoniae* possess a variant form of Ent, referred to as salmochelins (Fischbach et al., 2006), which escape the host cell Lcn2-mediated inhibition of salmochelin, leading to a growth advantage of the harmful bacteria over commensals. Thus, pathogenic gut microbes evolve different potential mechanisms to circumvent commensal-mediated colonization resistance and allow their establishment in the gut to colonize and cause infection.

### Nutrients as Signal Molecules

Apart from using nutrients as an energy source, pathogenic microbes use the lumen nutrient content or gut microbiota-derived molecules as metabolic signals of the host intestinal environment to adjust their activity accordingly. For example, EHEC uses fucose as a signaling molecule to regulate their metabolism and gene expression related to virulence and metabolic stimulus (Pacheco et al., 2012). EHEC possesses a fucose-sensing signaling transduction system, which is developed through the accumulation of pathogenicity island genes (virulence genes) that are acquired horizontally. It is a unique signaling system in EHEC and *C. rodentium* (Pacheco et al., 2012). In brief, the fucose-sensing signaling transduction system is basically composed of FusK and FusR components. FusK is a membrane-bounded histidine sensor kinase that undergoes autophosphorylation in the presence of fucose; later on, FusK transfers its phosphate to FusR. FusR is a response regulator of the fucose signaling system and acts as a transcription factor. Upon phosphorylation, FusR is activated, which causes the repression of the genes associated with fucose utilization in EHEC, thereby helping EHEC to avoid competing with commensal *E. coli* for this nutrient (Pacheco et al., 2012). In addition, EHEC avoids the unnecessary use of energy using FusR, which causes the repression of the genes associated with encoding the virulence machinery of the EHEC, which is a syringe-like apparatus also known as a type III secretion system (T3SS), which is used by bacteria EHEC to adhere to the host enterocytes and highjack the function of these cells (Pacheco et al., 2012). Therefore, EHEC utilizes fucose, a host-derived gut microbiota metabolic product, as a signal molecule that senses the intestinal lumen environment and adjusts its metabolism and virulence accordingly. Similarly, ethanolamine can also be used as a signal molecule by pathogenic bacteria such as EHEC and *S.* Typhimurium for the activation of their virulent gene expression (Kendall et al., 2012; Anderson et al., 2015). In addition to ethanolamine, *S.* Typhimurium exploits gut microbiota-derived hydrogen as a source of energy for its growth expansion at the initial stage of infection (Maier et al., 2013). Furthermore, EHEC and *C. rodentium* produce mucinases, which cleave the protein backbone of mucin-type glycoproteins, which are the main component of the host mucosa epithelial layer; by degrading this, these bacteria can access the lining of the epithelium (Szabady et al., 2009). The expression of these enzymes is enhanced with *B. thetaiotaomicron-*produced metabolites (Curtis et al., 2014). Actually, mucus is one of the major available sources of sugar in the gut colon, which is colonized by the bacteria EHEC and *C. rodentium*. As a consequence of the mucus layer devastation, a nutrient-poor environment is created near the epithelium, which is referred to as the gluconeogenic environment. Mice colonization with *B. thetaiotaomicron* profoundly changed the metabolic profile of the mouse colon by raising the level of organic acids such as succinate (Macy et al., 1978; Curtis et al., 2014; Ferreyra et al., 2014b). Moreover, a gluconeogenic environment is also characterized by an elevated level of several other metabolites such as lactate and glycerate (Curtis et al., 2014). EHEC and *C. rodentium* sense this gluconeogenic and succinate-rich environment of the colon through its transcriptional regulator Cra. Upon confirmation that they have gained access to the gut epithelium lining, these bacteria induce the expression of their secretory systems T3SSs (Curtis et al., 2014). Therefore, EHEC exploits the metabolic cues of the intestine lumen, which are produced by the microbiota, more specifically by *B. thetaiotaomicron*, and regulates its metabolism and virulence. Other pathogenic bacteria also use microbiota-produced metabolites as signaling molecules to adjust their metabolism and gene expression. For example, *C. difficile* utilizes the gut microbiota-produced succinate and transforms it into butyrate, thus gaining a growth advantage *in vivo* (Ferreyra et al., 2014b). In contrast, *C. difficile* mutant population is unable to catabolize succinate and fails to expand their growth in the gut due to the presence of *B. thetaiotaomicron* (Ferreyra et al., 2014b). More commonly, the gut microbiota produces SCFAs, which are more important metabolites that determine the interactions between the commensal microbiota and pathogenic bacteria in the intestine. The same as diet, the distribution, concentration, and composition of the SCFAs are distinct along with the different compartments of the intestine, and this difference may develop a different physiological environment, which may be sensed by the pathogenic bacteria signaling system as an environmental signal of the colon distinct region, thereby helping in the recognition of the niche by pathogenic bacteria. The most abundant SCFAs that are present in the gut are propionate, acetate, and butyrate. For example, the ileum part of the intestine is generally rich in acetate, having a concentration of 30 mM. This concentration of acetate enhances the expression of pathogenicity island 1 (SPI-1)-encoded T3SS (T3SS-1) of *S.* Typhimurium, which is involved in bacterium invasion in the host gut; therefore, the ileum region of the intestine is preferably colonized by *S.* Typhimurium (Carter and Collins, 1974). Conversely, the propionate and butyrate concentrations of 70 and 20 mM in the colon, respectively, repress the expression of T3SS-1-related genes (Lawhon et al., 2002), indicating that propionate and butyrate have an effect on the regulatory cascade of the T3SS-1 at various levels; however, the underlying mechanism of this regulation has not been unraveled yet. In the case of EHEC, the butyrate concentration found in the colon promotes the EHEC T3SS expression *via* post transcriptional activation of the Lrp, which is a transcriptional regulator in EHEC (Takao et al., 2014). Conversely, the exposure of the EHEC to the concentrations of acetate and propionate in the small intestine has not significantly affected the expression of genes related to the virulence of EHEC or EHEC T3SS (Takao et al., 2014). Diet is an important moderator of the healthy microbiome and has been known for its profound effect on the microbiota composition and SCFA concentration in the intestine (Kau et al., 2011). A fiber-rich diet leads to a higher production of butyrate by the intestinal microbiota, which enhances the expression of the host’s globotriaosylceramide, an enterocyte receptor for the Shiga toxin that is produced by EHEC (Zumbrun et al., 2013). In EHEC outbreaks, Shiga toxin is often associated with high morbidity and mortality and can lead to the development of a severe urinary tract complication known as hemolytic uremic syndrome (HUS) (Karmali et al., 1983). Consequently, animals using a fiber-rich diet may show more susceptibility to Shiga toxin than those using a fiber-poor or low-fiber diet and may develop a more severe disease (Zumbrun et al., 2013). In contrast, a high level of acetate has been identified with protection of the host from toxin-mediated disease. For example, certain species of commensal *Bifidobacteria* have been found to raise the level of acetate in the gut, which in turn helped in the prevention of Shiga toxin-mediated toxicity dissemination from the colon to the systemic circulation by promoting the intestinal epithelium barrier integrity and function (Fukuda et al., 2011). Thus, pathogenic bacteria require the exploitation of the microbiota-derived molecules both as signals and nutrients for successful colonization to cause infection in a host.

### The Use of the Host Immune Response by Pathogens for Their Advantage: Inflammation

Using the host immune response is another strategy used by pathogenic bacteria to have growth advantage over commensals, thereby inducing intestinal inflammation that prevents the survival of commensals in the gut environment. Most of the pathogenic gut microbes produce virulent factors such as toxins that induce gut inflammation. Pathogen-mediated intestinal inflammation or diarrhea substantially alters the balance of the gut microbial community, where the population of commensal microbiota decreases, which in turn increases the number and proliferation of existing or invading pathogens over commensals, thereby increasing the chance of pathogen colonization because of less competition (Lupp et al., 2007). A marked increase in *C. rodentium* growth was seen in the intestine of a DSS-induced mouse model of colitis; however, virulence factors are necessary for the colonization and proliferation of this bacterium, because the *ler* (a virulent factor gene) mutant has failed to get a survival advantage from DSS-induced intestinal inflammation to enhance their growth (Kamada et al., 2012). Similarly, *S.* Typhimurium also acquires growth advantage from self-induced intestinal inflammation. Normally, commensal microbiota releases an abundant amount of hydrogen sulfide (H_2_S), which is converted into thiosulfate $\left(S_{2}O_{3}^{2-}\right)$ by the host mucosa epithelium to avoid H_2_S-mediated host cell toxicity. During *S.* Typhimurium infection, a high level of recruited neutrophils and macrophages produces a huge pool of oxygen species that convert $S_{2}O_{3}^{2-}$ into tetrathionate $\left(S_{4}O_{6}^{2-}\right)$ (Levitt et al., 1999; Furne et al., 2001). Unlike commensal bacterial species, *S.* Typhimurium possesses the operon *ttrSR ttrBCA* that allows consuming $S_{4}O_{6}^{2-},$ resulting in a growth advantage of *S.* Typhimurium over commensal bacteria in the intestine during colitis (Winter et al., 2010). Furthermore, $S_{4}O_{6}^{2-}$ augments the growth of *S.* Typhimurium on ethanolamine (Thiennimitr et al., 2011). Likewise, other enteropathogenic bacteria including EHEC, EPEC, and *C. rodentium* may also benefit from intestinal inflammation. During intestinal inflammation, the gut mucosa tissue, migrated neutrophils, and macrophages having inducible nitric oxide synthetase enhance the production of nitrate (NO^3−^); as a result, the level of nitrate (NO^3−^) is raised in the intestine (Kolios et al., 2004; Reinders et al., 2007). The majority of the gut commensal microbiota are obligate anaerobes such as Bacteroidetes or Firmicutes that cannot use nitrate (NO^3−^) as an electron acceptor, but pathogenic bacteria, which are facultative anaerobes such as *E. coli*, express nitrate reductase enzymes and can use nitrate (NO^3−^) as an energy source for their growth, thus leading to a growth and survival advantage over anaerobic commensals in the inflamed intestine (Winter et al., 2013). Furthermore, the inflammatory environment of the host gut acts as a signal to trigger and enhance the expression of virulence factors and facilitate pathogen colonization and proliferation. For example, *P. aeruginosa*, a human opportunistic bacterial pathogen, causes nosocomial infection, which uses its outer membrane surface protein OprF and binds to the host immune factor interferon-γ (IFN-γ), thus inducing a quorum sensing-dependent virulence determinant type I *P. aeruginosa* (PA-I) lectin (Wu et al., 2005). This is how pathogens utilize the host inflammatory responses and have a growth advantage over commensals to promote their growth in host tissues.

## Intestinal Inflammation and Enteric Pathogen Outgrowth

Studies show that intestinal inflammation has always been associated with an imbalance of the gut microbiota in IBD patients as well as with experimental colitis models (Lupp et al., 2007; Garrett et al., 2010), which is characterized by a reduced diversity and abundance of obligate anaerobic bacteria such as Clostridia or Bacteroidia and an expansion of anaerobic, facultative bacteria such as Proteobacteria and other members of the Enterobacteriaceae (Seksik et al., 2003; Gophna et al., 2006; Baumgart et al., 2007; Walker et al., 2011; Gevers et al., 2014; Chiodini et al., 2015). These microbial changes during inflammation might reflect changes in the nutritional landscape of the gut environment, which is created by the host inflammatory responses. By inducing inflammation, the gut physiological environment and the available nutrient profiles are altered, which may lead to the inhibition of commensal bacteria and the proliferation of pathogenic bacteria due to the expression of unique metabolic pathways and virulence genes, which are absent in the commensals. For example, IL-22 is a cytokine abundant during *S.* Typhimurium infection that correlates with the high level of galactoside 2-α-l-fucosyltransferase 2 that promotes the α(1,2)-fucosylation of mucus carbohydrates, thus altering the level of fucose in the intestine lumen (Godinez et al., 2008; Pham et al., 2014; Pickard et al., 2014). The liberation of fucose from mucus carbohydrates leads to the activation of fucose-related genes in other members of the gut microbiota such as *E. coli* (Pickard et al., 2014). The rising level of the mucus-derived carbohydrate in the gut luminal nutrient content supports the growth of pathogenic bacteria; as a result, the composition of the gut microbiota is altered that may be implicated in disease occurrence (Sonnenburg et al., 2005; Ng et al., 2013). Similarly, during inflammation, the generation of reactive oxygen and nitrogen species and SCFA production alter the intestinal nutrient contents and the physiological environment which support pathogen growth. For example, the proinflammatory cytokine IFN-γ activates the mucosal epithelium dual oxidase 2, which causes the production of hydrogen peroxide (Harper et al., 2005). The gene *DUOX2* upregulation and their associative enzymes dual oxidase 2 have been found with an expansion of Proteobacteria in the gut microbiota of patients with Crohn’s disease and ulcerative colitis (Haberman et al., 2014). IFN-γ is also implicated in the expression of the gene *Nos2* (Salzman et al., 1996), which leads to the production of the inducible nitric oxide synthase that oxidizes l-arginine into nitric oxide (Palmer et al., 1988). Therefore, a high level of nitric oxide is present in the intestine of IBD patients (Lundberg et al., 1994; Singer et al., 1996; Enocksson et al., 2004). These radical species are transformed into non-toxic compounds such as nitrates and exist in elevated levels in the intestine of mice with colitis that can be used by members of the family Enterobacteriaceae such as *E. coli* and *S.* Typhimurium due to the presence of nitrate reductase enzymes, which convert nitrate into an electron receptor in a couple of reaction series, a process termed nitrate respiration, resulting in the expansion of the gut-resident pathobionts and obligate pathogens such as *E. coli* and *S.* Typhimurium, respectively (Lopez et al., 2012; Winter et al., 2013; Lopez et al., 2015). The generation of inflammatory factors during colitis creates a physiological niche in the lumen of the host gut that is enriched with pathogen-specific nutrients, which upregulate the growth of anaerobic facultative bacteria Enterobacteriaceae rather than obligate anaerobes (Winter et al., 2013). Consequently, pathogenic bacteria have growth advantages over the gut commensal bacteria population during intestinal inflammation; as a result, the gut microbiota community ecological interactions and microbe–microbe and microbe–host interactions are disturbed, which may be linked to enteric obligate pathogen colonization or resident pathobiont expansion and its associated intestinal infections. The respiratory nutrient-rich niche that results from the host immune-inflammatory responses is, therefore, a battlefield in which the gut bacteria commensals and pathogenic species fight for dominance using the diverse resources of nourishing and antimicrobial approaches.

## Microbiota-Targeted Therapies and Future Perspective

The idea of gut microbiota-targeting therapy has been used for years to prevent enteric pathogen infections; however, the lack of knowledge about the underlying mechanism of how commensals mediate colonization resistance and regulate resistance to pathogen colonization hampered the progression in the field. The current advancement in gut microbiome research revealed mechanistic insights into commensal–pathogen interaction, which may help in suggesting additional ways of pathogen prevention and eradication. For example, 16S rRNA and metagenome sequencing provide insights into the taxonomic composition and a detailed genetic capacity of the microbial community in the gut. Similarly, the use of germ-free animal models with emerging technologies, such as transcriptomics and laser-capture microdissection, has enabled the mechanistic associative studies of microbe–microbe and microbe–host interactions (Hooper et al., 2001). In addition, the emergence of new imaging quantitative technologies has enabled the site-specific microbial community localization and investigation of the complex microbial interaction within the gut and provided a high-resolution image of this complex chemistry landscape of the interactions between microbes and the host, which may facilitate the stage for intentionally informed manipulation of this chemistry with probiotic or prebiotic intervention to treat or prevent pathogen-associated diseases (Rath et al., 2012; Marcobal et al., 2013; Dorrestein et al., 2014; Bouslimani et al., 2015; Earle et al., 2015). EPEC and EHEC are the more common diarrhea-genic *E. coli* strains, responsible for the high rate of morbidity and mortality across the world each year (Kaper et al., 2004). In the mouse infectious model of *C. rodentium*, simple sugar that is released from the mucus carbohydrate regulates the ability of the gut microbial commensals such as commensal *E. coli* to outcompete the enteropathogenic strains of *E. coli* for the source of energy. The eradication of the EPEC and EHEC may be more efficient with commensal strains if the LEE-encoded virulence factors of the pathogen are targeted during the early stage of infections (Kamada et al., 2012). This approach may be effective in the eradication of not only the enteropathogens EPEC or EHEC but also other pathogens such as *C. difficile* and VRE. The overgrowth of *C. difficile* and VRE has been found as a leading cause of diarrhea and colitis among healthcare-associated infections (Rupnik et al., 2009; Arias and Murray, 2012), and a specific bacterial population has also been recognized with the clearance and eradication of *C. difficile* and VRE in the gut (Reeves et al., 2012; Ubeda et al., 2013). Although the underlying mechanism of action is unknown, it may be mediated through a direct mechanism such as competition for the limited sources of nutrients; thus, pathogenic bacteria may outcompete certain commensals having the same source of nutrients and energy. Therefore, the current understanding recommends the manipulation of the gut microbiota with the administration of probiotic strains metabolically related to EPEC or EHEC or prebiotic supplementation that could boost the growth of the gut-resident natural competitors, which may be an effective strategy to prevent these enteric infections. The emerging hybrid technology, metagenomics, and mathematical modeling may inform the development of precision microbiome reconstitution therapy (Buffie et al., 2015). Notably, the microbial transfer from a healthy donor to infected subjects, termed microbiota transplantation, has proven the efficacy of the gut microbiota-based therapy as an effective treatment approach in *C. difficile* infection which is refractory to chemotherapy (Reeves et al., 2012; Petrof et al., 2013; Nood et al., 2013). However, variation in the donor gut microbiota composition and the presence of possible potentially harmful microbes may limit the use of microbiota transplantation in the clinic setting. Therefore, identification and characterization of the gut microbiota-specific commensal species related to eradication and growth inhibition of pathogens are necessary; it may help in the formulation of the more targeted therapy against pathogen infection based on the use of a single commensal species or a combination of commensal species to treat the infection. In addition, understanding the metabolic pathways used by commensal bacteria for the prevention and eradication of pathogenic bacteria will help in the development of next-generation probiotics, where genetically modified commensal strains with enhanced anti-pathogenic capacity will be used to limit pathogen colonization and prevent infections more efficiently.

In these exciting movements, the progression of multidisciplinary research and the emergence of new technologies provide mechanistic insights into the interplay between the microbiota, host, and pathogens and offer a wide range of translational research opportunities for the biomedical research community to gain a molecular understanding of this cross-talk and transform it into new therapeutics options against infectious diseases.

## Conclusion

The current review of literature studies provides the latest insights into the interaction of microbiota in the gut. The first section particularly focuses on the mechanisms of commensal bacteria by which they mediate pathogen colonization resistance and eradicate pathogens from the gut environment. The second part is about the mechanisms of pathogenic bacteria and how pathogenic bacteria break up this resistance and colonize the gut and cause infections. Studies on the gut microbiota composition along the GIT indicated that their population in the intestine is dictated by nutrient availability, the physiological condition of the gut environment, and the gut microbial interaction within the community and with the host. In a healthy state, there is a delicate balance in the gut microbiota population where the commensals dominate over the pathobionts/pathogens and occupy all niches and nutrients along the intestine and restrain the pathobiont overgrowth and invaded or invading pathogen colonization by having efficient metabolic pathways that outcompete the access of pathogens for the limited nutrient resource in the intestine. Similarly, commensals activate the host immune response against pathogens to prevent their proliferation and change the gut physiological environment such as pH, which prevents pathogen virulence gene expression, which is essential for pathogen colonization. However, any interruption, commonly associated with antibiotic use or diet intervention, of this delicate balance between commensals and pathobionts ultimately results in the loss of commensal-mediated pathogen colonization resistance, which may lead to overpopulation of pathobionts/pathogens. Meanwhile, pathogens use their chemical machinery and express virulent factor genes, induce local inflammation, and convert the gut physiological environment into one that favors their growth and inhibits the commensal population, thus leading to infectious diseases. At present, the microbiome science is relatively quite young; therefore, it warrants further understanding of the gut microbial community interactions to decipher the complex relationship of commensal–pathogen interactions and the gut microbiota–host interactions, which may be helpful in the establishment of rational approaches to manage intestinal infectious diseases. In addition, host immunity has also been implicated in the gut microbiota regulation, in both commensal bacteria and pathogen-mediated inflammation, thus indicating a critical role of immune factors in determining the composition of the gut microbiota (Ubeda et al., 2012). Therefore, it is suggested that additional studies need to be undertaken to clarify the mechanisms on the host side by which they regulate and affect the gut microbiota during the host health and disease.

## Author Contributions

IK provided the study concept. CZ and HS provided guidance and resources. IK, YB, LZ, NU, HU, and SS searched and compiled the relevant literature. IK wrote the manuscript. HS reviewed the manuscript. CZ edited the manuscript and conducted the publishing process. The rest of the co-authors contributed equally as per the authorship policy of the article publication in peer review journals. All authors have read the journal policy and agreed to be accountable for all aspects of the work and have given the final approval of the manuscript to be published.

## Funding

This work was supported by the Jiangsu Science and Technology Major Project (BA2016036) and the Gansu Science and Technology Major Project (17ZD2FA009).

## Conflict of Interest

The authors declare that the research was conducted in the absence of any commercial or financial relationships that could be construed as a potential conflict of interest.

The reviewer MK declared a shared affiliation, though no other collaboration, with the authors.

## Publisher’s Note

All claims expressed in this article are solely those of the authors and do not necessarily represent those of their affiliated organizations, or those of the publisher, the editors and the reviewers. Any product that may be evaluated in this article, or claim that may be made by its manufacturer, is not guaranteed or endorsed by the publisher.

## References

[B1] Abt M. C. McKenney P. T. Pamer E. G. (2016). Clostridium Difficile Colitis: Pathogenesis and Host Defence. Nat. Rev. Microbiol. doi: 10.1038/nrmicro.2016.108 PMC5109054 27573580

[B2] Alavi S. Mitchell J. D. Cho J. Y. Liu R. Macbeth J. C. Hsiao A. (2020). Interpersonal Gut Microbiome Variation Drives Susceptibility and Resistance to Cholera Infection. Cell. doi: 10.1016/j.cell.2020.05.036 PMC7394201 32631492

[B3] Alverdy J. Chi H. S. Sheldon G. F. (1985). The Effect of Parenteral Nutrition on Gastrointestinal Immunity. The Importance of Enteral Stimulation. Ann. Surg. doi: 10.1097/00000658-198512000-00003 PMC1250998 3935061

[B4] Anderson C. J. Clark D. E. Adli M. Kendall M. M. (2015). Ethanolamine Signaling Promotes Salmonella Niche Recognition and Adaptation During Infection. PloS Pathog. doi: 10.1371/journal.ppat.1005278 PMC4643982 26565973

[B5] Arias C. A. Murray B. E. (2012). The Rise of the Enterococcus: Beyond Vancomycin Resistance. Nat. Rev. Microbiol. doi: 10.1038/nrmicro2761 PMC3621121 22421879

[B6] Autieri S. M. Lins J. J. Leatham M. P. Laux D. C. Conway T. Cohen P. S. (2007). L-Fucose Stimulates Utilization of D-Ribose by Escherichia Coli MG1655 Δfucao and E. Coli Nissle 1917 Δfucao Mutants in the Mouse Intestine and in M9 Minimal Medium. Infect. Immun. doi: 10.1128/IAI.00822-07 PMC2168271 17709419

[B7] Ayres J. S. Trinidad N. J. Vance R. E. (2012). Lethal Inflammasome Activation by a Multidrug-Resistant Pathobiont Upon Antibiotic Disruption of the Microbiota. Nat. Med. 18 (5), 799–8065. doi: 10.1038/nm.2729 22522562 PMC3472005

[B8] Baumgart M. Dogan B. Rishniw M. Weitzman G. Bosworth B. Yantiss R.. (2007). Culture Independent Analysis of Ileal Mucosa Reveals a Selective Increase in Invasive Escherichia Coli of Novel Phylogeny Relative to Depletion of Clostridiales in Crohn’s Disease Involving the Ileum. ISME J. doi: 10.1038/ismej.2007.52 18043660

[B9] Baümler A. J. Sperandio V. (2016). Interactions Between the Microbiota and Pathogenic Bacteria in the Gut. Nature. doi: 10.1038/nature18849 PMC5114849 27383983

[B10] Becattini S. Littmann E. R. Carter R. A. Kim S. G. Morjaria S. M. Ling L.. (2017). Commensal Microbes Provide First Line Defense Against Listeria Monocytogenes Infection. J. Exp. Med. doi: 10.1084/jem.20170495 PMC5502438 28588016

[B11] Benítez-Páez A. Gómez del Pugar E. M. López-Almela I. Moya-Pérez Á. Codoñer-Franch P. Sanz Y. (2020). Depletion of Blautia Species in the Microbiota of Obese Children Relates to Intestinal Inflammation and Metabolic Phenotype Worsening. MSystems. doi: 10.1128/msystems.00857-19 PMC7093825 32209719

[B12] Bertin Y. Girardeau J. P. Chaucheyras-Durand F. Lyan B. Pujos-Guillot E. Harel J.. (2011). Enterohaemorrhagic Escherichia Coli Gains a Competitive Advantage by Using Ethanolamine as a Nitrogen Source in the Bovine Intestinal Content. Environ. Microbiol. doi: 10.1111/j.1462-2920.2010.02334.x 20849446

[B13] Blander J. M. Longman R. S. Iliev I. D. Sonnenberg G. F. Artis D. (2017). Regulation of Inflammation by Microbiota Interactions With the Host. Nat. Immunol. doi: 10.1038/ni.3780 PMC5800875 28722709

[B14] Bohnhoff M. Drake B. L. Miller C. P. (1954). Effect of Streptomycin on Susceptibility of Intestinal Tract to Experimental Salmonella Infection. Proc. Soc. Exp. Biol. Med. doi: 10.3181/00379727-86-21030 13177610

[B15] Bokulich N. A. Chung J. Battaglia T. Henderson N. Jay M. Li H.. (2016). Antibiotics, Birth Mode, and Diet Shape Microbiome Maturation During Early Life. Sci. Trans. Med. doi: 10.1126/scitranslmed.aad7121 PMC5308924 27306664

[B16] Bouguénec C. L. Schouler C. (2011). Sugar Metabolism, an Additional Virulence Factor in Enterobacteria. Int. J. Med. Microbiol. doi: 10.1016/j.ijmm.2010.04.021 PMC7129668 20705507

[B17] Bourlioux P. Koletzko B. Guarner F. Braesco V. (2003). The Intestine and Its Microflora Are Partners for the Protection of the Host: Report on the Danone Symposium ‘The Intelligent Intestine,’ Held in Paris, June 14, 2002. Am. J. Clin. Nutr. doi: 10.1093/ajcn/78.4.675 14522724

[B18] Bouslimani A. Porto C. Rath C. M. Wang M. Guo Y. Gonzalez A.. (2015). Molecular Cartography of the Human Skin Surface in 3D. Proc. Natl. Acad. Sci. U. S. A. doi: 10.1073/pnas.1424409112 PMC4418856 25825778

[B19] Brandl K. Plitas G. Mihu C. N. Ubeda C. Jia T. Fleisher M.. (2008). Vancomycin-Resistant Enterococci Exploit Antibiotic-Induced Innate Immune Deficits. Nature. doi: 10.1038/nature07250 PMC2663337 18724361

[B20] Brinkman B. M. Becker A. Ayiseh R. B. Hildebrand F. Raes J. Huys G.. (2013). Gut Microbiota Affects Sensitivity to Acute DSS-Induced Colitis Independently of Host Genotype. Inflamm. Bowel Dis. doi: 10.1097/MIB.0b013e3182a8759a 24105395

[B21] Bry L. Falk P. G. Midtvedt T. Gordon J. I. (1996). A Model of Host-Microbial Interactions in an Open Mammalian Ecosystem. Science. doi: 10.1126/science.273.5280.1380 8703071

[B22] Buffie C. G. Bucci V. Stein R. R. McKenney P. T. Ling L. Gobourne A.. (2015). Precision Microbiome Reconstitution Restores Bile Acid Mediated Resistance to Clostridium Difficile. Nature. doi: 10.1038/nature13828 PMC4354891 25337874

[B23] Burgess S. L. Leslie J. L. Uddin J. Oakland D. N. Gilchrist C. Moreau G. B.. (2020). Gut Microbiome Communication With Bone Marrow Regulates Susceptibility to Amebiasis. J. Clin. Invest. doi: 10.1172/JCI133605 PMC7410058 32369444

[B24] Cameron E. A. Sperandio V. (2015). Frenemies: Signaling and Nutritional Integration in Pathogen-Microbiota-Host Interactions. Cell Host Microbe. doi: 10.1016/j.chom.2015.08.007 PMC4567707 26355214

[B25] Carter P. B. Collins F. M. (1974). The Route of Enteric Infection in Normal Mice. J. Exp. Med. doi: 10.1084/jem.139.5.1189 PMC2139651 4596512

[B26] Ceuppens S. Rajkovic A. Hamelink S. De Wiele T. V. Boon N. Uyttendaele M. (2012). Enterotoxin Production by Bacillus Cereus Under Gastrointestinal Conditions and Their Immunological Detection by Commercially Available Kits. Foodborne Pathog. Dis. doi: 10.1089/fpd.2012.1230 23237409

[B27] Cherrington C. A. Hinton M. Pearson G. R. Chopra I. (1991). Short-Chain Organic Acids at PH 5.0 Kill Escherichia Coli and Salmonella Spp. Without Causing Membrane Perturbation. J. Appl. Bacteriol. doi: 10.1111/j.1365-2672.1991.tb04442.x 1902205

[B28] Chiodini R. J. Dowd S. E. Chamberlin W. M. Galandiuk S. Davis B. Glassing A. (2015). Microbial Population Differentials Between Mucosal and Submucosal Intestinal Tissues in Advanced Crohn’s Disease of the Ileum. PloS One. doi: 10.1371/journal.pone.0134382 PMC4519195 26222621

[B29] Chow W. L. Lee Y. K. (2008). Free Fucose Is a Danger Signal to Human Intestinal Epithelial Cells. Br. J. Nutr. doi: 10.1017/S0007114507812062 17697405

[B30] Crosa J. H. Walsh C. T. (2002). Genetics and Assembly Line Enzymology of Siderophore Biosynthesis in Bacteria. Microbiol. Mol. Biol. Rev. doi: 10.1128/mmbr.66.2.223-249.2002 PMC120789 12040125

[B31] Curtis M. M. Hu Z. Klimko C. Narayanan S. Deberardinis R. Sperandio V. (2014). The Gut Commensal Bacteroides Thetaiotaomicron Exacerbates Enteric Infection Through Modification of the Metabolic Landscape. Cell Host Microbe. doi: 10.1016/j.chom.2014.11.005 PMC4269104 25498343

[B32] Dorrestein P. C. Mazmanian S. K. Knight R. (2014). Finding the Missing Links Among Metabolites, Microbes, and the Host. Immunity. doi: 10.1016/j.immuni.2014.05.015 PMC4503329 24950202

[B33] Dridi B. Raoult D. Drancourt M. (2011). Archaea as Emerging Organisms in Complex Human Microbiomes. Anaerobe. doi: 10.1016/j.anaerobe.2011.03.001 21420503

[B34] Ducarmon Q. R. Zwittink R. D. Hornung B. V. H. van Schaik W. Young V. B. Kuijper E. J. (2019). Gut Microbiota and Colonization Resistance Against Bacterial Enteric Infection. Microbiol. Mol. Biol. Rev. doi: 10.1128/mmbr.00007-19 PMC6710460 31167904

[B35] Earle K. A. Billings G. Sigal M. Lichtman J. S. Hansson G. C. Elias J. E.. (2015). Quantitative Imaging of Gut Microbiota Spatial Organization. Cell Host Microbe. doi: 10.1016/j.chom.2015.09.002 PMC4628835 26439864

[B36] Endt K. Stecher B. Chaffron S. Slack E. Tchitchek N. Benecke A.. (2010). The Microbiota Mediates Pathogen Clearance From the Gut Lumen After Non-Typhoidal Salmonella Diarrhea. PloS Pathog. doi: 10.1371/journal.ppat.1001097 PMC2936549 20844578

[B37] Enocksson A. Lundberg J. Weitzberg E. Norrby-Teglund A. Svenungsson B. (2004). Rectal Nitric Oxide Gas and Stool Cytokine Levels During the Course of Infectious Gastroenteritis. Clin. Diagn. Lab. Immunol. doi: 10.1128/CDLI.11.2.250-254.2004 PMC371199 15013971

[B38] Fabich A. J. Jones S. A. Chowdhury F. Z. Cernosek A. Anderson A. Smalley D.. (2008). Comparison of Carbon Nutrition for Pathogenic and Commensal Escherichia Coli Strains in the Mouse Intestine. Infect. Immun. doi: 10.1128/IAI.01386-07 PMC2258830 18180286

[B39] Fagarasan S. Kawamoto S. Kanagawa O. Suzuki K. (2010). Adaptive Immune Regulation in the Gut: T Cell-Dependent and T Cell-Independent IgA Synthesis. Annu. Rev. Immunol. doi: 10.1146/annurev-immunol-030409-101314 20192805

[B40] Fan D. Coughlin L. A. Neubauer M. M. Kim J. Kim M. S. Zhan X.. (2015). Activation of HIF-1α and LL-37 by Commensal Bacteria Inhibits Candida Albicans Colonization. Nat. Med. doi: 10.1038/nm.3871 PMC4496259 26053625

[B41] Fan Y. Pedersen O. (2021). Gut Microbiota in Human Metabolic Health and Disease. Nat. Rev. Microbiol. doi: 10.1038/s41579-020-0433-9 32887946

[B42] Feng Q. Chen W. D. Wang Y. D. (2018). Gut Microbiota: An Integral Moderator in Health and Disease. Front. Microbiol. doi: 10.3389/fmicb.2018.00151 PMC5826318 29515527

[B43] Ferreira R. B. R. Gill N. Willing B. P. Antunes L.C. M. Russell S. L. Croxen M. A.. (2011). The Intestinal Microbiota Plays a Role in Salmonella-Induced Colitis Independent of Pathogen Colonization. PloS One. doi: 10.1371/journal.pone.0020338 PMC3102097 21633507

[B44] Ferreyra J. A. Ng K. M. Sonnenburg J. L. (2014a). The Enteric Two-Step: Nutritional Strategies of Bacterial Pathogens Within the Gut. Cell. Microbiol. doi: 10.1111/cmi.12300 PMC4866598 24720567

[B45] Ferreyra J. A. Wu K. J. Hryckowian A. J. Bouley D. M. Weimer B. C. Sonnenburg J. L. (2014b). Gut Microbiota-Produced Succinate Promotes C. Difficile Infection After Antibiotic Treatment or Motility Disturbance. Cell Host Microbe. doi: 10.1016/j.chom.2014.11.003 PMC4859344 25498344

[B46] Fischbach M. A. Lin H. Liu D. R. Walsh C. T. (2006). How Pathogenic Bacteria Evade Mammalian Sabotage in the Battle for Iron. Nat. Chem. Biol. doi: 10.1038/nchembio771 16485005

[B47] Fischbach M. A. Sonnenburg J. L. (2011). Eating for Two: How Metabolism Establishes Interspecies Interactions in the Gut. Cell Host Microbe. doi: 10.1016/j.chom.2011.10.002 PMC3225337 22018234

[B48] Franchi L. Kamada N. Nakamura Y. Burberry A. Kuffa P. Suzuki S.. (2012). NLRC4-Driven Production of IL-1β Discriminates Between Pathogenic and Commensal Bacteria and Promotes Host Intestinal Defense. Nat. Immunol. doi: 10.1038/ni.2263 PMC3361590 22484733

[B49] Frantz A. L. Rogier E. W. Weber C. R. Shen L. Cohen D. A. Fenton L. A.. (2012). Targeted Deletion of MyD88 in Intestinal Epithelial Cells Results in Compromised Antibacterial Immunity Associated With Downregulation of Polymeric Immunoglobulin Receptor, Mucin-2, and Antibacterial Peptides. Mucosal Immunol. doi: 10.1038/mi.2012.23 PMC3422608 22491177

[B50] Fukuda S. Toh H. Hase K. Oshima K. Nakanishi Y. Yoshimura K.. (2011). Bifidobacteria Can Protect From Enteropathogenic Infection Through Production of Acetate. Nature. doi: 10.1038/nature09646 21270894

[B51] Fukuda S. Toh H. Taylor T. D. Ohno H. Hattori M. (2012). Acetate-Producing Bifidobacteria Protect the Host From Enteropathogenic Infection *via* Carbohydrate Transporters. Gut Microbes. doi: 10.4161/gmic.21214 22825494

[B52] Furne J. Springfield J. Koenig T. DeMaster E. Levitt M. D. (2001). Oxidation of Hydrogen Sulfide and Methanethiol to Thiosulfate by Rat Tissues: A Specialized Function of the Colonic Mucosa. Biochem. Pharmacol. doi: 10.1016/S0006-2952(01)00657-8 11389886

[B53] Gagliardi A. Totino V. Cacciotti F. Iebba V. Neroni B. Bonfiglio G.. (2018). Rebuilding the Gut Microbiota Ecosystem. Int. J. Environ. Res. Public Health. doi: 10.3390/ijerph15081679 PMC6121872 30087270

[B54] Gantois I. Ducatelle R. Pasmans F. Haesebrouck F. Hautefort I. Thompson A.. (2006). Butyrate Specifically Down-Regulates Salmonella Pathogenicity Island 1 Gene Expression. Appl. Environ. Microbiol. doi: 10.1128/AEM.72.1.946-949.2006 PMC1352287 16391141

[B55] Garrett W. S. Gallini C. A. Yatsunenko T. Michaud M. Dubois A. Delaney M. L.. (2010). Enterobacteriaceae Act in Concert With the Gut Microbiota to Induce Spontaneous and Maternally Transmitted Colitis. Cell Host Microbe. doi: 10.1016/j.chom.2010.08.004 PMC2952357 20833380

[B56] Garsin D. A. (2010). Ethanolamine Utilization in Bacterial Pathogens: Roles and Regulation. Nat. Rev. Microbiol. doi: 10.1038/nrmicro2334 PMC2950637 20234377

[B57] Gevers D. Kugathasan S. Denson L. A. Vázquez-Baeza Y. Van Treuren W. Ren B.. (2014). The Treatment-Naive Microbiome in New-Onset Crohn’s Disease. Cell Host Microbe. doi: 10.1016/j.chom.2014.02.005 PMC4059512 24629344

[B58] Ghosh S. Dai C. Brown K. Rajendiran E. Makarenko S. Baker J.. (2011). Colonic Microbiota Alters Host Susceptibility to Infectious Colitis by Modulating Inflammation, Redox Status, and Ion Transporter Gene Expression. Am. J. Physiol. Gastrointest. Liver Physiol. doi: 10.1152/ajpgi.00509.2010 21454446

[B59] Godinez I. Haneda T. Raffatellu M. George M. D. Paixão T. A. Rolán H. G.. (2008). T Cells Help to Amplify Inflammatory Responses Induced by Salmonella Enterica Serotype Typhimurium in the Intestinal Mucosa. Infect. Immun. doi: 10.1128/IAI.01691-07 PMC2346712 18347048

[B60] Gophna U. Sommerfeld K. Gophna S. Ford Doolittle W. Veldhuyzen Van Zanten S. J. O. (2006). Differences Between Tissue-Associated Intestinal Microfloras of Patients With Crohn’s Disease and Ulcerative Colitis. J. Clin. Microbiol. doi: 10.1128/JCM.01004-06 PMC1698347 16988016

[B61] Grünewald T. Ruf B. R. (2016). Clostridium Difficile Infections. Gynakol. Prax. doi: 10.7748/nop.22.4.13.s19

[B62] Haberman Y. Tickle T. L. Dexheimer P. J. Kim M. O. Tang D. Karns R.. (2014). Pediatric Crohn Disease Patients Exhibit Specific Ileal Transcriptome and Microbiome Signature. J. Clin. Invest. doi: 10.1172/JCI75436 PMC4109533 25003194

[B63] Hammami R. Fernandez B. Lacroix C. Fliss I. (2013). Anti-Infective Properties of Bacteriocins: An Update. Cell. Mol. Life Sci. doi: 10.1007/s00018-012-1202-3 PMC11113238 23109101

[B64] Harper R. W. Xu C. Eiserich J. P. Chen Y. Kao C. Y. Thai P.. (2005). Differential Regulation of Dual NADPH Oxidases/Peroxidases, Duox1 and Duox2, by Th1 and Th2 Cytokines in Respiratory Tract Epithelium. FEBS Lett. doi: 10.1016/j.febslet.2005.08.002 16111680

[B65] Hasegawa M. Kamada N. Jiao Y. Liu M. Z. Núñez G. Inohara N. (2012). Protective Role of Commensals Against Clostridium Difficile Infection *via* an IL-1β–Mediated Positive-Feedback Loop. J. Immunol. doi: 10.4049/jimmunol.1200821 PMC3752782 22888139

[B66] Hasegawa M. Osaka T. Tawaratsumida K. Yamazaki T. Tada H. Chen G. Y.. (2010). Transitions in Oral and Intestinal Microflora Composition and Innate Immune Receptor-Dependent Stimulation During Mouse Development Infect. Immun. doi: 10.1128/IAI.01043-09 PMC2812188 19933833

[B67] Hooper L. V. Wong M. H. Thelin A. Hansson L. Falk P. G. Gordon J. I. (2001). Molecular Analysis of Commensal Host-Microbial Relationships in the Intestine. Science. doi: 10.1126/science.291.5505.881 11157169

[B68] Hooper L. V. Xu J. Falk P. G. Midtvedt T. Gordon J. I. (1999). A Molecular Sensor That Allows a Gut Commensal to Control Its Nutrient Foundation in a Competitive Ecosystem. Proc. Natl. Acad. Sci. U. S. A. doi: 10.1073/pnas.96.17.9833 PMC22296 10449780

[B69] Hsiao A. Ahmed A.M.S. Subramanian S. Griffin N. W. Drewry L. L. Petri W. A.. (2014). Members of the Human Gut Microbiota Involved in Recovery From Vibrio Cholerae Infection. Nature. doi: 10.1038/nature13738 PMC4353411 25231861

[B70] Huttenhower C. Gevers D. Knight R. Abubucker S. Badger J. H. Chinwalla A. T.. (2012). Structure, Function and Diversity of the Healthy Human Microbiome. Nature. doi: 10.1038/nature11234 PMC3564958 22699609

[B71] Ivanov I. I. Atarashi K. Manel N. Brodie E. L. Shima T. Karaoz U.. (2009). Induction of Intestinal Th17 Cells by Segmented Filamentous Bacteria. Cell 139 (3), 485–498. doi: 10.1016/j.cell.2009.09.033 19836068 PMC2796826

[B72] Jacobson A. Lam L. Rajendram M. Tamburini F. Honeycutt J. Pham T.. (2018). A Gut Commensal-Produced Metabolite Mediates Colonization Resistance to Salmonella Infection. Cell Host Microbe. doi: 10.1016/j.chom.2018.07.002 PMC6223613 30057174

[B73] Jenior M. L. Leslie J. L. Young V. B. Schloss P. D. (2018). Clostridium Difficile Alters the Structure and Metabolism of Distinct Cecal Microbiomes During Initial Infection To Promote Sustained Colonization. MSphere. doi: 10.1128/msphere.00261-18 PMC6021602 29950381

[B74] Joseph B. Przybilla K. Stühler C. Schauer K. Slaghuis J. Fuchs T. M.. (2006). Identification of Listeria Monocytogenes Genes Contributing to Intracellular Replication by Expression Profiling and Mutant Screening. J. Bacteriol. doi: 10.1128/JB.188.2.556-568.2006 PMC1347271 16385046

[B75] Kamada N. Chen G. Y. Inohara N. Núñez G. (2013). Control of Pathogens and Pathobionts by the Gut Microbiota. Nat. Immunol. doi: 10.1038/ni.2608 PMC4083503 23778796

[B76] Kamada N. Kim Y. G. Sham H. P. Vallance B. A. Puente J. L. Martens E. C.. (2012). Regulated Virulence Controls the Ability of a Pathogen to Compete With the Gut Microbiota. Science. doi: 10.1126/science.1222195 PMC3439148 22582016

[B77] Kampmann C. Dicksved J. Engstrand L. Rautelin H. (2016). Composition of Human Faecal Microbiota in Resistance to Campylobacter Infection. Clin. Microbiol. Infect. doi: 10.1016/j.cmi.2015.09.004 26369602

[B78] Kaper J. B. Nataro J. P. Mobley H. L. T. (2004). Pathogenic Escherichia Coli. Nat. Rev. Microbiol. doi: 10.1038/nrmicro818 15040260

[B79] Karmali M. A. Petric M. Lim C. Fleming P. C. Steele B. T. (1983). Escherichia Coli Cytotoxin, Haemolytic-Uraemic Syndrome, And Haemorrhagic Colitis. Lancet. doi: 10.1016/S0140-6736(83)91167-4 6139632

[B80] Kau A. L. Ahern P. P. Griffin N. W. Goodman A. L. Gordon J. I. (2011). Human Nutrition, the Gut Microbiome and the Immune System. Nature. doi: 10.1038/nature10213 PMC3298082 21677749

[B81] Kendall M. M. Gruber C. C. Parker C. T. Sperandio V. (2012). Ethanolamine Controls Expression of Genes Encoding Components Involved in Interkingdom Signaling and Virulence in Enterohemorrhagic Escherichia Coli O157:H7. MBio. doi: 10.1128/mBio.00050-12 PMC3372972 22589288

[B82] Khan I. Ullah N. Zha L. Bai Y. Khan A. Zhao T.. (2019). Alteration of Gut Microbiota in Inflammatory Bowel Disease (IBD): Cause or Consequence? IBD Treatment Targeting the Gut Microbiome. Pathogens. doi: 10.3390/pathogens8030126 PMC6789542 31412603

[B83] Kim S. M. DeFazio J. R. Hyoju S. K. Sangani K. Keskey R. Krezalek M. A.. (2020). Fecal Microbiota Transplant Rescues Mice From Human Pathogen Mediated Sepsis by Restoring Systemic Immunity. Nat. Commun. doi: 10.1038/s41467-020-15545-w PMC7214422 32393794

[B84] Kinnebrew M. A. Ubeda C. Zenewicz L. A. Smith N. Flavell R. A. Pamer E. G. (2010). Bacterial Flagellin Stimulates Toll-Like Receptor 5-Dependent Defense Against Vancomycin-Resistant Enterococcus Infection. J. Infect. Dis. doi: 10.1086/650203 PMC2811237 20064069

[B85] Kiss E. A. Vonarbourg C. Kopfmann S. Hobeika E. Finke D. Esser C.. (2011). Natural Aryl Hydrocarbon Receptor Ligands Control Organogenesis of Intestinal Lymphoid Follicles. Science. doi: 10.1126/science.1214914 22033518

[B86] Kobayashi K. S. Chamaillard M. Ogura Y. Henegariu O. Inohara N. Nuñez G.. (2005). Nod2-Dependent Regulation of Innate and Adaptive Immunity in the Intestinal Tract. Science. doi: 10.1126/science.1104911 15692051

[B87] Koh A. Bäckhed F. (2020). From Association to Causality: The Role of the Gut Microbiota and Its Functional Products on Host Metabolism. Mol. Cell. doi: 10.1016/j.molcel.2020.03.005 32234490

[B88] Koh A. Vadder F. D. Kovatcheva-Datchary P. Bäckhed F. (2016). From Dietary Fiber to Host Physiology: Short-Chain Fatty Acids as Key Bacterial Metabolites. Cell. doi: 10.1016/j.cell.2016.05.041 27259147

[B89] Kolios G. Valatas V. Ward S. G. (2004). Nitric Oxide in Inflammatory Bowel Disease: A Universal Messenger in an Unsolved Puzzle. Immunology. doi: 10.1111/j.1365-2567.2004.01984.x PMC1782592 15554920

[B90] Korbel J. O. Doerks T. Jensen L. J. Perez-Iratxeta C. Kaczanowski S. Hooper S. D.. (2005). Systematic Association of Genes to Phenotypes by Genome and Literature Mining. PloS Biol. doi: 10.1371/journal.pbio.0030134 PMC1073694 15799710

[B91] Koropatkin N. M. Cameron E. A. Martens E. C. (2012). How Glycan Metabolism Shapes the Human Gut Microbiota. Nat. Rev. Microbiol. doi: 10.1038/nrmicro2746 PMC4005082 22491358

[B92] Lapidot Y. Amir A. Nosenko R. Uzan-Yulzari A. Veitsman E. Cohen-Ezra O.. (2020). Alterations in the Gut Microbiome in the Progression of Cirrhosis to Hepatocellular Carcinoma. MSystems. doi: 10.1128/msystems.00153-20 PMC7300357 32546668

[B93] Law R. J. Gur-Arie L. Rosenshine I. Finlay B. B. (2013). *In Vitro* and *In Vivo* Model Systems for Studying Enteropathogenic Escherichia Coli Infections. Cold Spring Harbor Perspect. Med. 3 (3). doi: 10.1101/cshperspect.a009977 PMC3579205 23457294

[B94] Lawhon S. D. Maurer R. Suyemoto M. Altier C. (2002). Intestinal Short-Chain Fatty Acids Alter Salmonella Typhimurium Invasion Gene Expression and Virulence Through BarA/SirA. Mol. Microbiol. doi: 10.1046/j.1365-2958.2002.03268.x 12453229

[B95] Lawley T. D. Clare S. Walker A. W. Goulding D. Stabler R. A. Croucher N.. (2009). Antibiotic Treatment of Clostridium Difficile Carrier Mice Triggers a Supershedder State, Spore-Mediated Transmission, and Severe Disease in Immunocompromised Hosts. Infect. Immun. doi: 10.1128/IAI.00558-09 PMC2737984 19564382

[B96] Lawley T. D. Walker A. W. (2013). Intestinal Colonization Resistance. Immunology. doi: 10.1111/j.1365-2567.2012.03616.x PMC3533696 23240815

[B97] Leatham M. P. Banerjee S. Autieri S. M. Mercado-Lubo R. Conway T. Cohen P. S. (2009). Precolonized Human Commensal Escherichia Coli Strains Serve as a Barrier to E. Coli O157:H7 Growth in the Streptomycin-Treated Mouse Intestine. Infect. Immun. doi: 10.1128/IAI.00059-09 PMC2708557 19364832

[B98] Leger A. J. St. Desai J. V. Drummond R. A. Kugadas A. Almaghrabi F. Silver P.. (2017). An Ocular Commensal Protects Against Corneal Infection by Driving an Interleukin-17 Response From Mucosal Γδ T Cells. Immunity. doi: 10.1016/j.immuni.2017.06.014 PMC5553552 28709803

[B99] Levitt M. D. Furne J. Springfield J. Suarez F. DeMaster E. (1999). Detoxification of Hydrogen Sulfide and Methanethiol in the Cecal Mucosa. J. Clin. Invest. doi: 10.1172/JCI7712 PMC408582 10525049

[B100] Ley R. E. Peterson D. A. Gordon J. I. (2006). Ecological and Evolutionary Forces Shaping Microbial Diversity in the Human Intestine. Cell. doi: 10.1016/j.cell.2006.02.017 16497592

[B101] Li E. Hamm C. M. Gulati A. S. Sartor R. B. Chen H. Wu X.. (2012). Inflammatory Bowel Diseases Phenotype, C. Difficile and NOD2 Genotype Are Associated With Shifts in Human Ileum Associated Microbial Composition. PloS One. doi: 10.1371/journal.pone.0026284 PMC3374607 22719818

[B102] Lim M. Y. You H. J. Yoon H. S. Kwon B. Lee J. Y. Lee S.. (2017). The Effect of Heritability and Host Genetics on the Gut Microbiota and Metabolic Syndrome. Gut. doi: 10.1136/gutjnl-2015-311326 27053630

[B103] Li J. Wang J. Jia H. Cai X. Zhong H. Feng Q.. (2014). An Integrated Catalog of Reference Genes in the Human Gut Microbiome. Nat. Biotechnol. doi: 10.1038/nbt.2942 24997786

[B104] Lopez C. A. Rivera-Chávez F. Byndloss M. X. Bäumler A. J. (2015). The Periplasmic Nitrate Reductase NapABC Supports Luminal Growth of Salmonella Enterica Serovar Typhimurium During Colitis. Infect. Immun. doi: 10.1128/IAI.00351-15 PMC4534643 26099579

[B105] Lopez C. A. Winter S. E. Rivera-Chávez F. Xavier M. N. Poon V. Nuccio S. P.. (2012). Phage-Mediated Acquisition of a Type III Secreted Effector Protein Boosts Growth of Salmonella by Nitrate Respiration. MBio. doi: 10.1128/mBio.00143-12 PMC3374392 22691391

[B106] Lundberg J. O. N. Weitzberg E. Lundberg J. M. Alving K. (1994). Intragastric Nitric Oxide Production in Humans: Measurements in Expelled Air. Gut. doi: 10.1136/gut.35.11.1543 PMC1375608 7828969

[B107] Lupp C. Robertson M. L. Wickham M. E. Sekirov I. Champion O. L. Gaynor E. C.. (2007). Host-Mediated Inflammation Disrupts the Intestinal Microbiota and Promotes the Overgrowth of Enterobacteriaceae. Cell Host Microbe. doi: 10.1016/j.chom.2007.06.010 18030708

[B108] Lv L.-X. Jiang H.-Y. Yan R. Li L. (2019). Interactions Between Gut Microbiota and Hosts and Their Role in Infectious Diseases. Infect. Microbes Dis. doi: 10.1097/im9.0000000000000001

[B109] Macpherson A. J. Geuking M. B. McCoy K. D. (2012). Homeland Security: IgA Immunity at the Frontiers of the Body. Trends Immunol. doi: 10.1016/j.it.2012.02.002 22410243

[B110] Macy J. M. Ljungdahl L. G. Gottschalk G. (1978). Pathway of Succinate and Propionate Formation in Bacteroides Fragilis. J. Bacteriol. doi: 10.1128/jb.134.1.84-91.1978 PMC222221 148460

[B111] Maier L. Vyas R. Cordova C. D. Lindsay H. Schmidt T. S. B. Brugiroux S.. (2013). Microbiota-Derived Hydrogen Fuels Salmonella Typhimurium Invasion of the Gut Ecosystem. Cell Host Microbe. doi: 10.1016/j.chom.2013.11.002 24331462

[B112] Maltby R. Leatham-Jensen M. P. Gibson T. Cohen P. S. Conway T. (2013). Nutritional Basis for Colonization Resistance by Human Commensal Escherichia Coli Strains HS and Nissle 1917 Against E. Coli O157:H7 in the Mouse Intestine. PloS One. doi: 10.1371/journal.pone.0053957 PMC3547972 23349773

[B113] Marcobal A. Kashyap P. C. Nelson T. A. Aronov P. A. Donia M. S. Spormann A.. (2013). A Metabolomic View of How the Human Gut Microbiota Impacts the Host Metabolome Using Humanized and Gnotobiotic Mice. ISME J. doi: 10.1038/ismej.2013.89 PMC3965317 23739052

[B114] Marteyn B. West N. P. Browning D. F. Cole J. A. Shaw J. G. Palm F.. (2010). Modulation of Shigella Virulence in Response to Available Oxygen *In Vivo* . Nature. doi: 10.1038/nature08970 PMC3750455 20436458

[B115] Martinez-Guryn K. Hubert N. Frazier K. Urlass S. Musch M. W. Ojeda P.. (2018). Small Intestine Microbiota Regulate Host Digestive and Absorptive Adaptive Responses to Dietary Lipids. Cell Host Microbe. doi: 10.1016/j.chom.2018.03.011 PMC5912695 29649441

[B116] Matamoros S. Gras-Leguen C. Le Vacon F. Potel G. de la Cochetiere M. F. (2013). Development of Intestinal Microbiota in Infants and Its Impact on Health. Trends Microbiol. doi: 10.1016/j.tim.2012.12.001 23332725

[B117] McKenney P. T. Pamer E. G. (2015). From Hype to Hope: The Gut Microbiota in Enteric Infectious Disease. Cell. doi: 10.1016/j.cell.2015.11.032 PMC4672394 26638069

[B118] Modi S. R. Collins J. J. Relman D. A. (2014). Antibiotics and the Gut Microbiota. J. Clin. Invest. 124 (10), 4212–4185. doi: 10.1172/JCI72333 PMC4191029 25271726

[B119] Momose Y. (2008b). Effect of Organic Acids on Inhibition of Escherichia Coli O157:H7 Colonization in Gnotobiotic Mice Associated With Infant Intestinal Microbiota. Antonie Van Leeuwenhoek Int. J. Gen. Mol. Microbiol. doi: 10.1007/s10482-007-9188-9 17674138

[B120] Momose Y. Hirayama K. Itoh K. (2008a). Competition for Proline Between Indigenous Escherichia Coli and E. Coli O157:H7 in Gnotobiotic Mice Associated With Infant Intestinal Microbiota and Its Contribution to the Colonization Resistance Against E. Coli O157:H7. Antonie Van Leeuwenhoek Int. J. Gen. Mol. Microbiol. doi: 10.1007/s10482-008-9222-6 18247153

[B121] Mullineaux-Sanders C. Suez J. Elinav E. Frankel G. (2018). Sieving Through Gut Models of Colonization Resistance. Nat. Microbiol. 3 (2), 132–405. doi: 10.1038/s41564-017-0095-1 29358683

[B122] Ng K. M. Ferreyra J. A. Higginbottom S. K. Lynch J. B. Kashyap P. C. Gopinath S.. (2013). Microbiota-Liberated Host Sugars Facilitate Post-Antibiotic Expansion of Enteric Pathogens. Nature. doi: 10.1038/nature12503 PMC3825626 23995682

[B123] Ng J. Hirota S. A. Gross O. Li Y. Ulke-Lemee A. Potentier M. S.. (2010). Clostridium Difficile Toxin-Induced Inflammation and Intestinal Injury Are Mediated by the Inflammasome. Gastroenterology. doi: 10.1053/j.gastro.2010.04.005 20398664

[B124] Nood E. V. Vrieze A. Nieuwdorp M. Fuentes S. Zoetendal E. G. de Vos W. M.. (2013). Duodenal Infusion of Donor Feces for Recurrent Clostridium Difficile. N Engl. J. Med. doi: 10.1056/nejmoa1205037 23323867

[B125] Oh P. L. Martínez I. Sun Y. Walter J. Peterson D. A. Mercer D. F. (2012). Characterization of the Ileal Microbiota in Rejecting and Nonrejecting Recipients of Small Bowel Transplants. Am. J. Transplant. doi: 10.1111/j.1600-6143.2011.03860.x 22152019

[B126] Pacheco A. R. Munera D. Waldor M. K. Sperandio V. Ritchie J. M. (2012). Fucose Sensing Regulates Bacterial Intestinal Colonization. Nature. doi: 10.1038/nature11623 PMC3518558 23160491

[B127] Pacheco A. R. Sperandio V. (2015). “Enteric Pathogens Exploit the Microbiota-Generated Nutritional Environment of the Gut,” in Metabolism and Bacterial Pathogenesis. doi: 10.1128/microbiolspec.mbp-0001-2014 PMC5070792 26185079

[B128] Palmer R. M. J. Rees D. D. Ashton D. S. Moncada S. (1988). L-Arginine Is the Physiological Precursor for the Formation of Nitric Oxide in Endothelium-Dependent Relaxation. Biochem. Biophys. Res. Commun. doi: 10.1016/S0006-291X(88)81362-7 3390182

[B129] Parhi L. Alon-Maimon T. Sol A. Nejman D. Shhadeh A. Fainsod-Levi T.. (2020). Breast Cancer Colonization by Fusobacterium Nucleatum Accelerates Tumor Growth and Metastatic Progression. Nat. Commun. doi: 10.1038/s41467-020-16967-2 PMC7320135 32591509

[B130] Pavia A. T. Shipman L. D. Wells J. G. Puhr N. D. Smith J. D. McKinley T. W.. (1990). Epidemiologic Evidence That Prior Antimicrobial Exposure Decreases Resistance to Infection by Antimicrobial-Sensitive Salmonella. J. Infect. Dis. doi: 10.1093/infdis/161.2.255 2299207

[B131] Pedicord V. A. Lockhart A. A. K. Rangan K. J. Craig J. W. Loschko J. Rogoz A.. (2016). Exploiting a Host-Commensal Interaction to Promote Intestinal Barrier Function and Enteric Pathogen Tolerance. Sci. Immunol. doi: 10.1126/sciimmunol.aai7732 PMC5453653 28580440

[B132] Perna N. T. Plunkett G. Burland V. Mau B. Glasner J. D. Rose D. J.. (2001). Genome Sequence of Enterohaemorrhagic Escherichia Coli O157:H7. Nature. doi: 10.1038/35054089 11206551

[B133] Petnicki-Ocwieja T. Hrncir T. Liu Y. J. Biswas A. Hudcovic T. Tlaskalova-Hogenova H.. (2009). Nod2 Is Required for the Regulation of Commensal Microbiota in the Intestine. Proc. Natl. Acad. Sci. U. S. A. doi: 10.1073/pnas.0907722106 PMC2747201 19805227

[B134] Petrof E. O. Gloor G. B. Vanner S. J. Weese S. J. Carter D. Daigneault M. C.. (2013). Stool Substitute Transplant Therapy for the Eradication of Clostridium Difficile Infection: ‘Repoopulating’ the Gut. Microbiome. doi: 10.1186/2049-2618-1-3 PMC3869191 24467987

[B135] Pham T. A. N. Clare S. Goulding D. Arasteh J. M. Stares M. D. Browne H. P.. (2014). Epithelial IL-22ra1-Mediated Fucosylation Promotes Intestinal Colonization Resistance to an Opportunistic Pathogen. Cell Host Microbe. doi: 10.1016/j.chom.2014.08.017 PMC4190086 25263220

[B136] Pickard J. M. Maurice C. F. Kinnebrew M. A. Abt M. C. Schenten D. Golovkina T. V.. (2014). Rapid Fucosylation of Intestinal Epithelium Sustains Host-Commensal Symbiosis in Sickness. Nature. doi: 10.1038/nature13823 PMC4214913 25274297

[B137] Pickard J. M. Núñez G. (2019). Pathogen Colonization Resistance in the Gut and Its Manipulation for Improved Health. Am. J. Pathol. doi: 10.1016/j.ajpath.2019.03.003 PMC6617533 31100210

[B138] Piewngam P. Zheng Y. Nguyen T. H. Dickey S. W. Joo H. S. Villaruz A. E.. (2018). Pathogen Elimination by Probiotic Bacillus *via* Signalling Interference. Nature. doi: 10.1038/s41586-018-0616-y PMC6202238 30305736

[B139] Pridmore R. D. Berger B. Desiere F. Vilanova D. Barretto C. Pittet A. C.. (2004). The Genome Sequence of the Probiotic Intestinal Bacterium Lactobacillus Johnsionii NCC 533. Proc. Natl. Acad. Sci. U. S. A. doi: 10.1073/pnas.0307327101 PMC356981 14983040

[B140] Qiu J. Heller J. J. Guo X. Chen Z. M. E. Fish K. Fu Y. X.. (2012). The Aryl Hydrocarbon Receptor Regulates Gut Immunity Through Modulation of Innate Lymphoid Cells. Immunity. doi: 10.1016/j.immuni.2011.11.011 PMC3268875 22177117

[B141] Rakoff-Nahoum S. Coyne M. J. Comstock L. E. (2014). An Ecological Network of Polysaccharide Utilization Among Human Intestinal Symbionts. Curr. Biol doi: 10.1016/j.cub.2013.10.077 PMC3924574 24332541

[B142] Raman R. Thomas R. G. Weiner M. W. Jack C. R. Ernstrom K. Aisen P. S.. (2005). Diversity of the Human Intestinal Microbial Flora. Science.

[B143] Rath C. M. Alexandrov T. Higginbottom S. K. Song J. Milla M. E. Fischbach M. A.. (2012). Molecular Analysis of Model Gut Microbiotas by Imaging Mass Spectrometry and Nanodesorption Electrospray Ionization Reveals Dietary Metabolite Transformations. Anal. Chem. doi: 10.1021/ac302039u PMC3711173 23009651

[B144] Reeves A. E. Koenigsknecht M. J. Bergin I. L. Young V. B. (2012). Suppression of Clostridium Difficile in the Gastrointestinal Tracts of Germfree Mice Inoculated With a Murine Isolate From the Family Lachnospiraceae. Infect. Immun. doi: 10.1128/IAI.00647-12 PMC3486043 22890996

[B145] Reinders C. I. Jonkers D. Jansson E. Å. Stockbrügger R. W. Stobberingh E. E. Hellström P. M.. (2007). Rectal Nitric Oxide and Fecal Calprotectin in Inflammatory Bowel Disease. Scand. J. Gastroenterol. doi: 10.1080/00365520701320505 17852876

[B146] Rivera-Chávez F. Bäumler A. J. (2015). The Pyromaniac Inside You: Salmonella Metabolism in the Host Gut. Annu. Rev. Microbiol. doi: 10.1146/annurev-micro-091014-104108 26002180

[B147] Rivera-Chávez F. Zhang L. F. Faber F. Lopez C. A. Byndloss M. X. Olsan E. E.. (2016). Depletion of Butyrate-Producing Clostridia From the Gut Microbiota Drives an Aerobic Luminal Expansion of Salmonella. Cell Host Microbe. doi: 10.1016/j.chom.2016.03.004 PMC4832419 27078066

[B148] Rolhion N. Chassaing B. (2016). When Pathogenic Bacteria Meet the Intestinal Microbiota. Philos. Trans. R. Soc. B Biol. Sci. doi: 10.1098/rstb.2015.0504 PMC5052746 27672153

[B149] Rupnik M. Wilcox M. H. Gerding D. N. (2009). Clostridium Difficile Infection: New Developments in Epidemiology and Pathogenesis. Nat. Rev. Microbiol. doi: 10.1038/nrmicro2164 19528959

[B150] Ryan F. J. Ahern A. M. Fitzgerald R. S. Laserna-Mendieta E. J. Power E. M. Clooney A. G.. (2020). Colonic Microbiota Is Associated With Inflammation and Host Epigenomic Alterations in Inflammatory Bowel Disease. Nat. Commun. doi: 10.1038/s41467-020-15342-5 PMC7089947 32251296

[B151] Salzman A. L. Denenberg A. G. Ueta I. O’Connor M. Linn S. C. Szabó C. (1996). Induction and Activity of Nitric Oxide Synthase in Cultured Human Intestinal Epithelial Monolayers. Am. J. Physiol. Gastrointest. Liver Physiol. doi: 10.1152/ajpgi.1996.270.4.g565 8928785

[B152] Salzman N. H. Hung K. Haribhai D. Chu H. Karlsson-Sjöberg J. Amir E.. (2010). Enteric Defensins Are Essential Regulators of Intestinal Microbial Ecology. Nat. Immunol. doi: 10.1038/ni.1825 PMC2795796 19855381

[B153] Sanos S. L. Bui V. L. Mortha A. Oberle K. Heners C. Johner C.. (2009). RORγt and Commensal Microflora Are Required for the Differentiation of Mucosal Interleukin 22-Producing NKp46+ Cells. Nat. Immunol. doi: 10.1038/ni.1684 PMC4217274 19029903

[B154] Sassone-Corsi M. Raffatellu M. (2015). No Vacancy: How Beneficial Microbes Cooperate With Immunity To Provide Colonization Resistance to Pathogens. J. Immunol. doi: 10.4049/jimmunol.1403169 PMC4402713 25888704

[B155] Satoh-Takayama N. Vosshenrich C. A. J. Lesjean-Pottier S. Sawa S. Lochner M. Rattis F.. (2008). Microbial Flora Drives Interleukin 22 Production in Intestinal NKp46+ Cells That Provide Innate Mucosal Immune Defense. Immunity. doi: 10.1016/j.immuni.2008.11.001 19084435

[B156] Schamberger G. P. Diez-Gonzalez F. (2002). Selection of Recently Isolated Colicinogenic Escherichia Coli Strains Inhibitory to Escherichia Coli O157:H7. J. Food Prot. doi: 10.4315/0362-028X-65.9.1381 12233846

[B157] Schauer D. B. Falkow S. (1993). Attaching and Effacing Locus of a Citrobacter Freundii Biotype That Causes Transmissible Murine Colonic Hyperplasia. Infect. Immun. 61 (6), 2486–2492. doi: 10.1128/iai.61.6.2486-2492.1993 8500884 PMC280873

[B158] Schroeder B. O. Bäckhed F. (2016). Signals From the Gut Microbiota to Distant Organs in Physiology and Disease. Nat. Med. doi: 10.1038/nm.4185 27711063

[B159] Scott S. A. Fu J. Chang P. V. (2020). Microbial Tryptophan Metabolites Regulate Gut Barrier Function *via* the Aryl Hydrocarbon Receptor. Proc. Natl. Acad. Sci. U. S. A. doi: 10.1073/pnas.2000047117 PMC7431026 32719140

[B160] Sekirov I. Russell S. L. Antunes L.C. M. Finlay B. B. (2010). Gut Microbiota in Health and Disease. Physiol. Rev. doi: 10.1152/physrev.00045.2009 20664075

[B161] Sekirov I. Tam N. M. Jogova M. Robertson M. L. Li Y. Lupp C.. (2008). Antibiotic-Induced Perturbations of the Intestinal Microbiota Alter Host Susceptibility to Enteric Infection. Infect. Immun. doi: 10.1128/IAI.00319-08 PMC2546810 18678663

[B162] Seksik P. Rigottier-Gois L. Gramet G. Sutren M. Pochart P. Marteau P.. (2003). Alterations of the Dominant Faecal Bacterial Groups in Patients With Crohn’s Disease of the Colon. Gut 52 (2), 237–242. doi: 10.1136/gut.52.2.237 12524406 PMC1774977

[B163] Sender R. Fuchs S. Milo R. (2016). Are We Really Vastly Outnumbered? Revisiting the Ratio of Bacterial to Host Cells in Humans. Cell. doi: 10.1016/j.cell.2016.01.013 26824647

[B164] Shin R. Suzuki M. Morishita Y. (2002). Influence of Intestinal Anaerobes and Organic Acids on the Growth of Enterohaemorrhagic Escherichia Coli O157:H7. J. Med. Microbiol. doi: 10.1099/0022-1317-51-3-201 11871614

[B165] Singer I. I. Kawka D. W. Scott S. Weidner J. R. Mumford R. A. Riehl T. E.. (1996). Expression of Inducible Nitric Oxide Synthase and Nitrotyrosine in Colonic Epithelium in Inflammatory Bowel Disease. Gastroenterology. doi: 10.1016/S0016-5085(96)70055-0 8831582

[B166] Sonnenburg J. L. Bäckhed F. (2016). Diet-Microbiota Interactions as Moderators of Human Metabolism. Nature. doi: 10.1038/nature18846 PMC5991619 27383980

[B167] Sonnenburg J. L. Xu J. Leip D. D. Chen C. H. Westover B. P. Weatherford J.. (2005). Glycan Foraging *In Vivo* by an Intestine-Adapted Bacterial Symbiont. Science. doi: 10.1126/science.1109051 15790854

[B168] Sorbara M. T. Pamer E. G. (2019). Correction: Interbacterial Mechanisms of Colonization Resistance and the Strategies Pathogens Use to Overcome Them (Mucosal Immunology, (2019), 12, 1, (1-9), 10.1038/S41385-018-0053-0). Mucosal Immunol. doi: 10.1038/s41385-019-0151-7 30796335

[B169] Sprinz H. Kundel D. W. Dammin G. J. Horowitz R. E. Schneider H. Formal S. B. (1961). The Response of the Germfree Guinea Pig to Oral Bacterial Challenge With Escherichia Coli and Shigella Flexneri. Am. J. Pathol. PMC1942415 13915950

[B170] Stecher B. Robbiani R. Walker A. W. Westendorf A. M. Barthel M. Kremer M.. (2007). Salmonella Enterica Serovar Typhimurium Exploits Inflammation to Compete With the Intestinal Microbiota. PloS Biol. doi: 10.1371/journal.pbio.0050244 PMC1951780 17760501

[B171] Steed A. L. Christophi G. P. Kaiko G. E. Sun L. Goodwin V. M. Jain U.. (2017). The Microbial Metabolite Desaminotyrosine Protects From Influenza Through Type I Interferon. Science. doi: 10.1126/science.aam5336 PMC5753406 28774928

[B172] Strugnell R. A. Wijburg O. L. C. (2010). The Role of Secretory Antibodies in Infection Immunity. Nat. Rev. Microbiol. doi: 10.1038/nrmicro2384 20694027

[B173] Suzuki K. Maruya M. Kawamoto S. Sitnik K. Kitamura H. Agace W. W.. (2010). The Sensing of Environmental Stimuli by Follicular Dendritic Cells Promotes Immunoglobulin A Generation in the Gut. Immunity. doi: 10.1016/j.immuni.2010.07.003 20643338

[B174] Szabady R. L. Lokuta M. A. Walters K. B. Huttenlocher A. Welch R. A. (2009). Modulation of Neutrophil Function by a Secreted Mucinase of Escherichia Coli O157:H7. PloS Pathog. doi: 10.1371/journal.ppat.1000320 PMC2642718 19247439

[B175] Takao M. Yen H. Tobe T. (2014). LeuO Enhances Butyrate-Induced Virulence Expression Through a Positive Regulatory Loop in Enterohaemorrhagic Escherichia Coli. Mol. Microbiol. doi: 10.1111/mmi.12737 25069663

[B176] Thanissery R. Winston J. A. Theriot C. M. (2017). Inhibition of Spore Germination, Growth, and Toxin Activity of Clinically Relevant C. Difficile Strains by Gut Microbiota Derived Secondary Bile Acids. Anaerobe. doi: 10.1016/j.anaerobe.2017.03.004 PMC5466893 28279860

[B177] The Human Microbiome Project Consortium (2012). Structure, Function and Diversity of the Healthy Human Microbiome The Human Microbiome Project Consortium*. Nature. 10.1038/nature11234 PMC3564958 22699609

[B178] Theriot C. M. Koenigsknecht M. J. Carlson P. E. Hatton G. E. Nelson A. M. Li B.. (2014). Antibiotic-Induced Shifts in the Mouse Gut Microbiome and Metabolome Increase Susceptibility to Clostridium Difficile Infection. Nat. Commun. doi: 10.1038/ncomms4114 PMC3950275 24445449

[B179] Theriot C. M. Young V. B. (2015). Interactions Between the Gastrointestinal Microbiome and Clostridium Difficile. Annu. Rev. Microbiol. doi: 10.1146/annurev-micro-091014-104115 PMC4892173 26488281

[B180] Thiennimitr P. Winter S. E. Winter M. G. Xavier M. N. Tolstikov V. Huseby D. L.. (2011). Intestinal Inflammation Allows Salmonella to Use Ethanolamine to Compete With the Microbiota. Proc. Natl. Acad. Sci. U. S. A. doi: 10.1073/pnas.1107857108 PMC3198331 21969563

[B181] Tierney B. T. Yang Z. Luber J. M. Beaudin M. Wibowo M. C. Baek C.. (2019). The Landscape of Genetic Content in the Gut and Oral Human Microbiome. Cell Host Microbe. doi: 10.1016/j.chom.2019.07.008 PMC6716383 31415755

[B182] Turnbaugh P. J. Bäckhed F. Fulton L. Gordon J. I. (2008). Diet-Induced Obesity Is Linked to Marked But Reversible Alterations in the Mouse Distal Gut Microbiome. Cell Host Microbe. doi: 10.1016/j.chom.2008.02.015 PMC3687783 18407065

[B183] Turovskiy Y. Sutyak Noll K. Chikindas M. L. (2011). The Aetiology of Bacterial Vaginosis. J. Appl. Microbiol. doi: 10.1111/j.1365-2672.2011.04977.x PMC3072448 21332897

[B184] Ubeda C. Bucci V. Caballero S. Djukovic A. Toussaint N. C. Equinda M.. (2013). Intestinal Microbiota Containing Barnesiella Species Cures Vancomycin-Resistant Enterococcus Faecium Colonization. Infect. Immun. doi: 10.1128/IAI.01197-12 PMC3584866 23319552

[B185] Ubeda C. Lipuma L. Gobourne A. Viale A. Leiner I. Equinda M.. (2012). Familial Transmission Rather Than Defective Innate Immunity Shapes the Distinct Intestinal Microbiota of TLR-Deficient Mice. J. Exp. Med. doi: 10.1084/jem.20120504 PMC3409501 22826298

[B186] Vaishnava S. Behrendt C. L. Ismail A. S. Eckmann L. Hooper L. V. (2008). Paneth Cells Directly Sense Gut Commensals and Maintain Homeostasis at the Intestinal Host-Microbial Interface. Proc. Natl. Acad. Sci. U. S. A. doi: 10.1073/pnas.0808723105 PMC2603261 19075245

[B187] Vaishnava S. Yamamoto M. Severson K. M. Ruhn K. A. Yu X. Koren O.. (2011). The Antibacterial Lectin RegIIIγ Promotes the Spatial Segregation of Microbiota and Host in the Intestine. Science. doi: 10.1126/science.1209791 PMC3321924 21998396

[B188] Waaij D.V. D. Berghuis-de Vries J. M. Lekkerkerk-Van Der Wees J. E. C. (1971). Colonization Resistance of the Digestive Tract in Conventional and Antibiotic-Treated Mice. J. Hyg. doi: 10.1017/S0022172400021653 PMC2130899 4999450

[B189] Walker A. W. Sanderson J. D. Churcher C. Parkes G. C. Hudspith B. N. Rayment N.. (2011). High-Throughput Clone Library Analysis of the Mucosa-Associated Microbiota Reveals Dysbiosis and Differences Between Inflamed and Non-Inflamed Regions of the Intestine in Inflammatory Bowel Disease. BMC Microbiol. doi: 10.1186/1471-2180-11-7 PMC3032643 21219646

[B190] Willing B. Halfvarson J. Dicksved J. Rosenquist M. Järnerot G. Engstrand L.. (2009). Twin Studies Reveal Specific Imbalances in the Mucosa-Associated Microbiota of Patients With Ileal Crohn’s Disease. Inflamm. Bowel Dis. doi: 10.1002/ibd.20783 19023901

[B191] Willing B. P. Vacharaksa A. Croxen M. Thanachayanont T. Brett Finlay B. (2011). Altering Host Resistance to Infections Through Microbial Transplantation. PloS One. doi: 10.1371/journal.pone.0026988 PMC3203939 22046427

[B192] Winkler E. S. Shrihari S. Hykes B. L. Handley S. A. Andhey P. S. Huang Y. J. S.. (2020). The Intestinal Microbiome Restricts Alphavirus Infection and Dissemination Through a Bile Acid-Type I IFN Signaling Axis. Cell. doi: 10.1016/j.cell.2020.06.029 PMC7483520 32668198

[B193] Winter S. E. Thiennimitr P. Winter M. G. Butler B. P. Huseby D. L. Crawford R. W.. (2010). Gut Inflammation Provides a Respiratory Electron Acceptor for Salmonella. Nature. doi: 10.1038/nature09415 PMC2946174 20864996

[B194] Winter S. E. Winter M. G. Xavier M. N. Thiennimitr P. Poon V. Keestra A. M.. (2013). Host-Derived Nitrate Boosts Growth of E. Coli in the Inflamed Gut. Science. doi: 10.1126/science.1232467 PMC4004111 23393266

[B195] Wlodarska M. Willing B. P. Bravo D. M. Finlay B. B. (2015). Phytonutrient Diet Supplementation Promotes Beneficial Clostridia Species and Intestinal Mucus Secretion Resulting in Protection Against Enteric Infection. Sci. Rep. doi: 10.1038/srep09253 PMC4365398 25787310

[B196] Wu L. Estrada O. Zaborina O. Bains M. Shen L. Kohler J. E.. (2005). Microbiology: Recognition of Host Immune Activation by Pseudomonas Aeruginosa. Science. doi: 10.1126/science.1112422 16051797

[B197] Yilmaz B. Portugal S. Tran T. M. Gozzelino R. Ramos S. Gomes J.. (2014). Gut Microbiota Elicits a Protective Immune Response Against Malaria Transmission. Cell. doi: 10.1016/j.cell.2014.10.053 PMC4261137 25480293

[B198] Zachar Z. Savage D. C. (1979). Microbial Interference and Colonization of the Murine Gastrointestinal Tract by Listeria Monocytogenes. Infect. Immun. doi: 10.1128/iai.23.1.168-174.1979 PMC550704 106003

[B199] Zeng M. Y. Cisalpino D. Varadarajan S. Hellman J. Warren H. S. Cascalho M.. (2016). Gut Microbiota-Induced Immunoglobulin G Controls Systemic Infection by Symbiotic Bacteria and Pathogens. Immunity. doi: 10.1016/j.immuni.2016.02.006 PMC4794373 26944199

[B200] Zheng D. Liwinski T. Elinav E. (2020). Interaction Between Microbiota and Immunity in Health and Disease. Cell Res. doi: 10.1038/s41422-020-0332-7 PMC7264227 32433595

[B201] Zheng Y. Valdez P. A. Danilenko D. M. Hu Y. Sa S. M. Gong Q.. (2008). Interleukin-22 Mediates Early Host Defense Against Attaching and Effacing Bacterial Pathogens. Nat. Med. doi: 10.1038/nm1720 18264109

[B202] Zumbrun S. D. Melton-Celsa A. R. Smith M. A. Gilbreath J. J. Merrell D. S. O’Brien A. D. (2013). Dietary Choice Affects Shiga Toxin-Producing Escherichia Coli (STEC) O157:H7 Colonization and Disease. Proc. Natl. Acad. Sci. U. S. A. doi: 10.1073/pnas.1222014110 PMC3677460 23690602

